# Organic Electronic Platform for Real‐Time Phenotypic Screening of Extracellular‐Vesicle‐Driven Breast Cancer Metastasis

**DOI:** 10.1002/adhm.202301194

**Published:** 2023-05-21

**Authors:** Walther C. Traberg, Johana Uribe, Victor Druet, Adel Hama, Chrysanthi‐Maria Moysidou, Miriam Huerta, Reece McCoy, Daniel Hayward, Achilleas Savva, Amaury M. R. Genovese, Suraj Pavagada, Zixuan Lu, Anil Koklu, Anna‐Maria Pappa, Rebecca Fitzgerald, Sahika Inal, Susan Daniel, Róisín M. Owens

**Affiliations:** ^1^ Department of Chemical Engineering and Biotechnology University of Cambridge Cambridge CB3 0AS UK; ^2^ Robert F. Smith School of Chemical and Biomolecular Engineering Cornell University Olin Hall Ithaca NY 14853 USA; ^3^ Biological and Environmental Sciences and Engineering Division King Abdullah University of Science and Technology (KAUST) Thuwal 3955 Kingdom of Saudi Arabia; ^4^ Early Cancer Institute University of Cambridge Hutchison Research Centre Cambridge CB2 0XZ UK; ^5^ Healthcare Innovation Engineering Center Khalifa University Abu Dhabi PO Box 127788 United Arab Emirates; ^6^ Department of Biomedical Engineering Khalifa University of Science and Technology Abu Dhabi PO Box 127788 United Arab Emirates

**Keywords:** breast cancer metastasis, drug screening, exosomes, extracellular vesicles, organic electronics

## Abstract

Tumor‐derived extracellular vesicles (TEVs) induce the epithelial‐to‐mesenchymal transition (EMT) in nonmalignant cells to promote invasion and cancer metastasis, representing a novel therapeutic target in a field severely lacking in efficacious antimetastasis treatments. However, scalable technologies that allow continuous, multiparametric monitoring for identifying metastasis inhibitors are absent. Here, the development of a functional phenotypic screening platform based on organic electrochemical transistors (OECTs) for real‐time, noninvasive monitoring of TEV‐induced EMT and screening of antimetastatic drugs is reported. TEVs derived from the triple‐negative breast cancer cell line MDA‐MB‐231 induce EMT in nonmalignant breast epithelial cells (MCF10A) over a nine‐day period, recapitulating a model of invasive ductal carcinoma metastasis. Immunoblot analysis and immunofluorescence imaging confirm the EMT status of TEV‐treated cells, while dual optical and electrical readouts of cell phenotype are obtained using OECTs. Further, heparin, a competitive inhibitor of cell surface receptors, is identified as an effective blocker of TEV‐induced EMT. Together, these results demonstrate the utility of the platform for TEV‐targeted drug discovery, allowing for facile modeling of the transient drug response using electrical measurements, and provide proof of concept that inhibitors of TEV function have potential as antimetastatic drug candidates.

## Graphical abstract

Dual, electronic and optical monitoring of epithelial to mesenchymal transition (EMT) caused by tumor‐derived extracellular vesicles, an early, key stage in tumor progression, is made possible using the organic electrochemical transistor based on the conducting polymer poly(3,4‐ethylenedioxythiophene) doped with poly(styrene sulfonate). Continuous electrical monitoring enables screening of new strategies to block EMT.

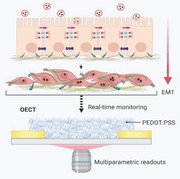

## 1 Introduction

According to the World Health Organisation (WHO), cancer is a leading cause of death worldwide, accounting for nearly 10 million deaths, or one in six deaths in 2020 (https://gco.iarc.fr/today). The primary cause of cancer‐related mortalities is widespread metastases, which are difficult to treat with therapeutic intervention.^[^ ^1^ ^]^ Meanwhile, drug development is a slow and costly process,^[^ ^2^ ^]^ with a failure rate of 96.6% for Food and Drug Administration (FDA) approvals in clinical trials for oncology,^[^ ^3^ ^]^ primarily due to insufficient efficacy (48%/55%) or safety (25%/14%; phase II/III).^[^ ^4^ ^]^ The development of reliable preclinical drug screening technologies and disease models is therefore a healthcare imperative. Although metastasis is a hallmark of cancer, it remains poorly understood,^[^ ^1^, ^5^ ^]^ in part due to challenges in characterizing its spatial and temporal progression.^[^ ^5^ ^]^ Recently, nanosized particles secreted by tumor cells implicated in intercellular communication, called extracellular vesicles (EVs), have been found to play a pivotal role in metastasis.^[^ ^6^, ^7^ ^]^ Tumor‐derived EVs (TEVs) carry functional biomolecules that confer oncogenic potential^[^ ^8^ ^]^ and induce malignant transformation in recipient cells.^[^ ^9^ ^]^ TEVs drive a process called the epithelial‐to‐mesenchymal transition (EMT), which is a central component of metastasis.^[^ ^7^, ^10^, ^11^, ^12^, ^13^ ^]^ EMT is a reversible process by which epithelial cells at the invasive front of carcinoma acquire a mesenchymal phenotype^[^ ^14^ ^]^ and develop the ability to migrate, invade,^[^ ^15^ ^]^ and disseminate via circulation to form metastases in distal organs.^[^ ^16^ ^]^ Targeting TEVs with therapeutic intervention could represent a novel avenue for preventing EMT, and thereby metastasis.

Phenotypic drug discovery approaches incorporating cell‐based disease models have the potential to address the incompletely understood complexity of diseases, compared to target‐based drug discovery. Although phenotypic screening can be an attractive proposition for efficiently identifying functionally active hits that lead to first‐in‐class drugs,^[^ ^17^ ^]^ current readouts suffer limitations. These include relying on sensing technologies that are invasive; perturbing the system of study (e.g., omics), produce discrete data, and/or scale poorly (e.g., microscopy imaging).^[^ ^18^, ^19^ ^]^ This makes it difficult to investigate the temporal relationship between measured pharmacodynamic response and pharmacokinetics for quantitative translation to in vivo outcomes.^[^ ^20^ ^]^ Moreover, utilizing low‐throughput technologies to screen thousands of potential hits during the early stages of drug discovery is cost‐ and time‐prohibitive.^[^ ^21^ ^]^ Thus, there is a pressing need to integrate disease‐relevant cell‐based models with novel, scalable sensing technologies that produce unbiased high‐content and multiparametric readouts, ideally in a continuous manner, without disrupting the native state of the system of study.^[^ ^17^ ^]^ Here, we present a platform based on organic bioelectronic technology that fulfills these requirements.

The field of organic electronics has progressed enormously since the initial discovery of conducting polymers^[^ ^22^ ^]^ and has recently gained widespread applications in biomedical research.^[^ ^23^, ^24^, ^25^ ^]^ One example is the organic electrochemical transistor (OECT), a device that has been integrated with in vitro models of varying complexities spanning from plasma membranes to 3D complex cell cultures,^[^ ^26^, ^27^, ^28^, ^29^, ^30^, ^31^ ^]^ possible due to its widely reported biocompatibility.^[^ ^32^ ^]^ OECTs comprise a three‐terminal configuration consisting of source and drain electrodes, on either side of a conducting polymer channel, and a gate electrode that controls the channel conductivity. Belonging to the broader class of “electrolyte gated transistors,” OECTs operate in direct contact with an electrolyte.^[^ ^33^ ^]^ Their operation relies on ions being “pushed” into the conducting polymer channel upon application of a gate bias, changing its doping state, hence its conductivity.^[^ ^34^ ^]^ The coupling between ionic (from the gate electrode) and electronic (from the drain electrode) charges within the entire volume of the conducting polymer channel is a particularly interesting feature of this device configuration. This setup produces record high signal amplification values that scale with the volume of the channel.^[^ ^35^ ^]^ Hence, OECTs have been widely considered as especially promising biological signal amplifiers^[^ ^36^ ^]^ without necessitating the incorporation of external circuits. The conducting polymer poly(3,4‐ethylenedioxythiophene doped with poly(styrene sulfonate) (PEDOT:PSS) is the material of choice in OECTs due to its excellent stability and easy processability.^[^ ^37^ ^]^ When in contact with aqueous biological environments, PEDOT:PSS swells, providing a biocompatible hydrogel‐like interface for cells.^[^ ^38^ ^]^ Another key feature of PEDOT:PSS is its optical transparency (when deposited as a thin film), making it compatible with microscopy, thereby enabling simultaneous optical and electrical readouts,^[^ ^26^, ^29^, ^30^ ^]^ pivotal in biological assays. PEDOT:PSS‐based OECTs have been used to monitor cell coverage and differentiation, as well as epithelial barrier integrity both in Transwell format and with cells adhered directly on the device surface.^[^ ^26^, ^28^, ^39^, ^40^ ^]^ The latter involves the growth of a monolayer of cells on the channel and the gate, which introduces a barrier for ion motion in the electrolyte (cell culture media), directly affecting the transistor output and in effect using the OECT as an impedance biosensor.^[^ ^28^, ^41^ ^]^ Monitoring tight junction modulation, nephrotoxicity, cancer invasion, wound healing, and toxicology^[^ ^27^, ^28^, ^42^, ^43^, ^44^ ^]^ are only some of the various application areas where impedance‐based OECTs interfaced with cell cultures have been successfully used for biomedical research, although biological validation of the electrical readouts has been limited in scope to date.

**Figure 1:**
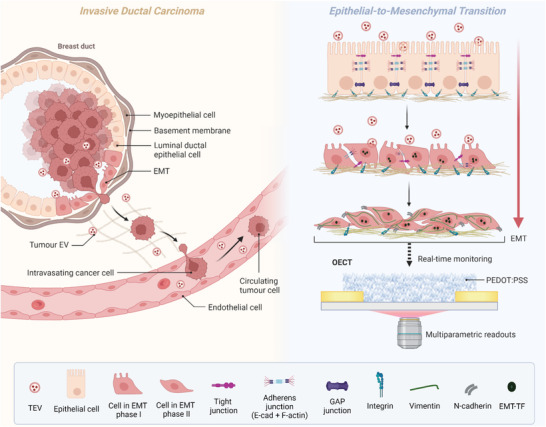
A model to recapitulate invasive ductal carcinoma integrated with an OECT platform for monitoring TEV‐induced EMT. EMT has been implicated in the initiation of metastasis, as epithelial cells at the invasive front of carcinomas acquire migratory and invasive properties to break through the basement membrane and disseminate via circulation to form metastases in distal organs.^[^ ^16^ ^]^ Postinternalization, recipient cells exhibit physiological changes associated with alterations of their transcriptome and proteome.^[^ ^48^, ^49^ ^]^ TEV exposure results in increased expression of several mesenchymal markers, including vimentin and TWIST1,^[^ ^50^, ^51^ ^]^ and decreased expression of epithelial markers, including reciprocal changes in E‐cadherin and N‐cadherin expression^[^ ^48^ ^]^ – the so‐called cadherin switch. Functionally, TEVs enhance the migratory and invasive properties of recipient cells.^[^ ^52^, ^53^ ^]^ By integrating a highly relevant model of breast cancer metastasis associated EMT on OECTs, this malignant process can be monitored in real time with multiparametric readouts and the effect of TEV‐targeting drugs can be assessed.

Capitalizing on these key attributes, here we have interfaced OECTs with a cell‐based EMT‐disease model to address current issues facing sensing technologies and provide proof of concept for organic‐electronic‐based phenotypic drug screening. OECTs were integrated with MCF10A cells; a nontumorigenic, mammary cell line,^[^ ^45^, ^46^ ^]^ and exposed to TEVs derived from MDA‐MB‐231 cells; a highly aggressive and invasive triple‐negative breast cancer (TNBC) cell line,^[^ ^47^ ^]^ to recapitulate a model of invasive ductal carcinoma (**Figure** **1** and Discussion S1 (Supporting Information)). We demonstrate the ability of the OECT to monitor TEV‐induced EMT in real time, without compromising compatibility with conventional end‐point assays. We leverage the capabilities of OECT devices to provide a continuous, label‐free, nondisruptive, and quantitative measure of EMT in vitro, using electrical measurements as a novel functional readout of a metastatic process, as well as of TEV function. We correlate the electrical readouts with molecular biology and optical microscopy techniques to validate the OECT‐based measurements. Finally, we demonstrate that we can dynamically monitor therapeutic response and find that heparin, a competitive inhibitor of cell surface receptors, effectively abrogates TEV‐induced EMT in MCF10A cells, showcasing the drug screening capabilities of our platform.

## 2 Characterization of Extracellular Vesicles

MCF10A cells were exposed to either MDA–TEVs or EVs derived from nontumorigenic human embryonic kidney (HEK)‐293 cells (HEK–EVs), to demonstrate that the associated EMT‐inducing effect was caused specifically by TEVs, rather than EVs in general – a so‐called specificity check. EVs were isolated by ultracentrifugation, but without further purification to avoid compromising particle yield and may therefore be described as crude EVs. The propensity for coisolates when using ultracentrifugation (UC) is not inherently problematic, as in vivo EVs exist together with other molecules that may contribute to their function/stability or even expedite their mode of action.^[^ ^54^ ^]^ It is possibly even less physiologically relevant to study EVs in complete isolation from any other coisolates. EVs were characterized following the guidelines of the International Society for Extracellular Vesicle for EV research,^[^ ^54^ ^]^ which include EV quantification by particle number and total protein content, detection of expected and not expected protein markers present in enriched EV samples, and single vesicle analysis. We used total protein content for quantifying isolate amounts, regardless of their specific composition; albeit prone to biases and errors, nanoparticle tracking analysis (NTA) particle quantifications maintain a very similar ratio in the two preparations, reinforcing the hypothesis that similar protein contents correspond to similar particle counts in both sources. In the cell experiments, we subjected individual cells to the same number of particles, based on the above.

**Figure 2:**
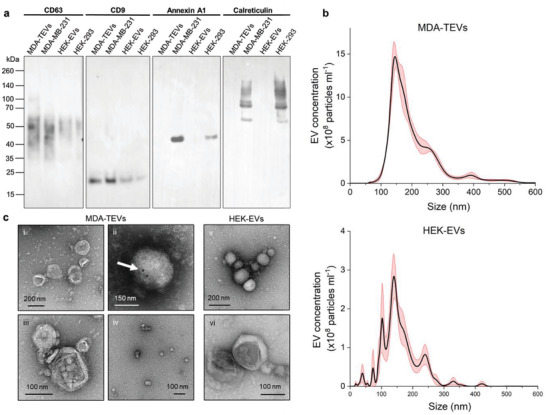
EV physical and biochemical characterization. a) Immunoblots of MDA‐MB‐231 and HEK‐293 whole‐cell lysates and (T)EV samples. Proteins were separated on SDS‐PAGE gels and membranes were blotted with indicated antibodies. CD63: smeared band between 35–60 kDa (multiple glycosylated forms influence gel migration, therefore CD63 appears as a smear in the 30–60 kDa range, as expected^[^ ^60^ ^]^); CD9, predicted: 25 kDa; annexin A1, predicted: 38 kDa; calreticulin, predicted: 55 kDa (other bands may indicate presence of dimers and multimers). Unedited immunoblots are available in Figure S1 (Supporting Information). b) Concentration and size distribution, calculated by NTA, of EVs (mean ± s.e.m.; *n* = 2 per EV type), with peak particle sizes of 153 ± 5.1 and 112 ± 12.7 nm for MDA–TEVs and HEK–EVs, respectively. c) Representative negative stain TEM images of indicated EVs (*n* = 3). i) MDA–TEVs. Scale bar, 200 nm. ii) MDA–TEV with 20 nm gold nanoparticles conjugated via CD63 antibody (white arrow). Scale bar, 150 nm. iii) Close‐up of MDA–TEV. Scale bar, 100 nm. iv) Smaller MDA–TEVs. Scale bar, 100 nm. v) HEK–EVs. Scale bar, 200 nm. vi) Close‐up of HEK–EV. Scale bar, 100 nm.

Samples were enriched in the classic EV protein biomarkers CD63 and CD9 and did not contain calreticulin; a protein located in the endoplasmic reticulum, nor annexin A1; a microvesicle (150–1000 nm sized EVs) biomarker^[^ ^55^ ^]^ (**Figure** **2**a), as expected. NTA revealed heterogenous EV populations, with peak particle sizes of 153 ± 5.1 and 112 ± 12.7 nm for MDA–TEVs and HEK–EVs, respectively (Figure 2b). Although such EVs may qualify as exosomes, without confirmation of their endosomal origin, they should be characterized as CD63/CD9‐positive EVs.^[^ ^54^ ^]^ Interestingly, EV particle concentration varied by a factor of >6 between the cell types, with 145 ± 2.3 × 10^9^ MDA–TEVs and 23 ± 0.8 × 10^9^ HEK–EVs derived from the same number of cells. This is consistent with reports that cancer cells produce an overabundance of EVs compared to healthy cells,^[^ ^56^, ^57^ ^]^ with their biogenesis possibly enhanced by hypoxic conditions prevalent in the tumor microenvironment.^[^ ^58^ ^]^ This difference was equally reflected in the total protein content of intact vesicles (**Table** **1**). EVs had particle zeta potentials of −12.6 ± 0.62 mV for MDA–TEVs and −6.2 ± 0.25 mV for HEK–EVs, as expected.^[^ ^59^ ^]^ EVs displayed characteristic spherical and cup‐shaped morphologies on transmission electron microscopy (TEM) images, and size heterogeneity is apparent with vesicles ranging from 50 to 200 nm in diameter (Figure 2c). Immunogold labeling of MDA–TEVs was performed to confirm the presence of CD63 on the EV's surface (Figure 2c(ii)).

**Table 1:** EV characterization data. Mean ± s.e.m.; values calculated from measurements of EVs collected from *n* = 2 and 3 different cell passages for each cell line for NTA/zeta potential and protein concentration, measured using a Qubit 4 fluorometer, respectively

EV	Size [nm]	*ζ*‐potential [mV]	Concentration [particles mL^−1^]	Protein content [µg mL^−1^]	Ratio of microgram protein to billion particles
MDA–TEV	153 ± 5.1	−12.6 ± 0.62	145 ± 2.3 × 10^9^	2411 ± 23.2	16.6
HEK–EV	112 ± 12.7	−6.2 ± 0.25	23 ± 0.8 × 10^9^	350 ± 3.5	15.4

## 3 Continuous OECT‐Based Monitoring of TEV‐Induced EMT

Current cell‐based screening approaches and accompanying sensing technologies suffer from limitations that result in the loss of useful information and hamper the development of therapeutic interventions against EMT. Thereto, very few of these techniques are amenable to multiparametric readouts without running experiments in parallel. End‐point assays that yield discrete data require multiple time points to be incorporated into the experimental design to track the dynamic changes that occur during EMT. While functional assays, such as migration or invasion assays, allow functional changes to be assessed, these are often limited to optical techniques, which are inherently low throughout and semiquantitative at best. To address this, we developed a novel functional readout of EMT using dynamic impedance‐based monitoring on OECTs to probe changes in epithelial cell barrier integrity resulting from malignant transformation. The electrical “tightness” of a cellular monolayer reflects the ionic conductance of the paracellular and transcellular pathways in an epithelial monolayer.^[^ ^61^ ^]^ Although cells grown on Transwell may have enhanced differentiation due to perception of cues from basal media, the resistance of cells adhered on substrates has previously been measured and correlates with barrier cell differentiation to epithelial phenotypes.^[^ ^39^ ^]^ Additionally, the integration of cells directly onto an electroactive substrate allows for better compatibility with optical characterization. Resistance of the cell layer is a quantitative measure of barrier integrity and permeability, and, as such, can be correlated to the EMT status of cells (Figure 1). We postulated that the ability to continuously and noninvasively monitor cell phenotype during EMT could help shed light on the incidence and implication of EMT hybrid states in metastasis initiation and elucidate the timescales of TEV function; a crucial insight for developing potent therapeutic interventions (see Discussion S2 in the Supporting Information).

To achieve this, we had to adapt our OECT platform to ensure that it was compatible with long‐term monitoring studies and could be integrated seamlessly with the EMT model. Previous studies had functionalized the OECT surface with collagen type I to promote cell adhesion,^[^ ^27^, ^43^ ^]^ however, collagen type I promotes EMT and tumor invasion in cancer cells.^[^ ^62^, ^63^ ^]^ Therefore, to avoid any confounding variables, we instead incubated the OECTs with normal MCF10A culture media overnight prior to cell seeding to promote adherence of cells. Moreover, the commonly used external Ag/AgCl electrodes^[^ ^43^, ^44^ ^]^ are cytotoxic^[^ ^64^, ^65^, ^66^ ^]^ and thus inappropriate for long‐term studies. Instead, we opted to use a planar gold gate electrode to ensure that the devices produced stable outputs over extended periods of time. The absence of any external electrode also considerably reduced the risk of contamination and improved data acquisition times.

**Figure 3:**
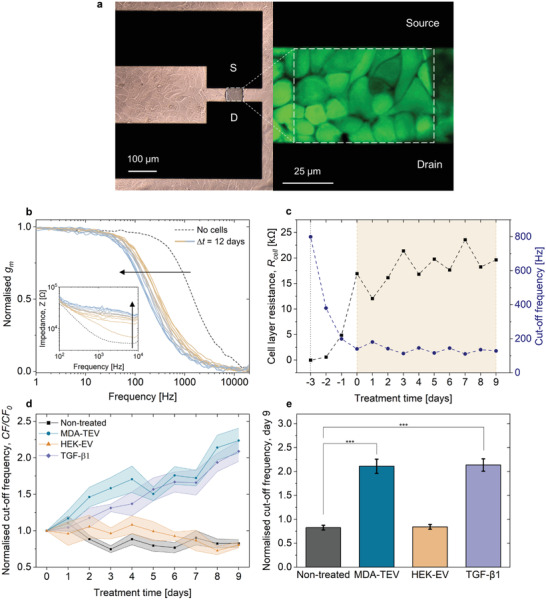
Real‐time, OECT‐based monitoring of EMT. a) Brightfield image of MCF10A cells on an OECT and zoomed‐in view of the channel with cells stained with calcein AM (green‐LIVE). The black boxes: source and drain electrodes; darkened area between them: PEDOT:PSS channel. b) Typical time evolution of the OECT frequency‐dependent response as cells grow and form a confluent layer under normal culture conditions (nontreated). Inset graph shows the evolution of the impedance for nontreated cells. c) Typical in‐line monitoring of the cutoff frequency, as derived from the transconductance versus frequency plot; and the cell layer resistance (*R*_cell_), as derived from the impedance versus frequency plot for nontreated cells. The vertical dotted line represents the baseline without cells on the device and the shaded area represents the equivalent treatment window. Representative cutoff frequency and *R*_cell_ plots are available for all conditions in Figure S4 (Supporting Information). d) Cutoff frequency normalized to day 0 (treatment start day) over time for the four experimental conditions: nontreated; 200 µg MDA–TEVs; 185 µg HEK–EVs; and 10 ng mL^−1^ TGF‐*β*1 (mean ± s.e.m.; indicated by lightly colored areas; *n* = 3 and 2 for nontreated/MDA–TEV and TGF‐*β*1/HEK–EV, respectively; see the Experimental Section for explanation on calculation of EV dose). Data points before treatment day 0 are omitted for clarity. e) Normalized cutoff frequency on treatment day 9 compared across the four conditions (mean ± s.e.m.). Two‐way ANOVA. *** *p* ≤0.001.

The addition of a cell layer between the channel (**Figure** **3**a) and the gate affects the OECT gating efficiency, rendering the device slower to respond to a given applied gate bias.^[^ ^30^ ^]^ The modulation of the device response time serves as a measure of the epithelial barrier integrity and is defined as the cutoff frequency, corresponding to the frequency at 70.7% of the maximum transconductance.^[^ ^39^ ^]^ The cutoff frequency is a figure of merit that defines the regimes of high and low ionic transport.^[^ ^36^ ^]^ Another important readout relevant to cells undergoing EMT can be derived from impedance measurements of the OECT channel as a function of frequency. As shown in the inset of Figure 3b, the impedance of the channels increases for frequencies over ≈2 kHz. At this frequency range, the impedance of our system is dominated by the resistance of the cell layer with the effect of capacitance being negligible.^[^ ^67^ ^]^ Therefore, we can directly correlate this increase of impedance with the cell layer resistance.

To assess the ability of MDA–TEVs to promote EMT in MCF10A cells, four experimental conditions were tested: 1) nontreated cells cultured with normal medium; 2) cells treated with MDA–TEVs representing the treatment condition; 3) cells treated with transforming growth factor beta 1 (TGF‐*β*1) representing a positive control, as TGF‐*β*1 is commonly used to induce EMT in epithelial cells;^[^ ^68^ ^]^ and 4) cells treated with HEK–EVs representing a negative control and a specificity check. The culture medium (normal or supplemented with MDA–TEVs, HEK–EVs, or TGF‐*β*1) was refreshed every 48 h, thereby exposing the cells to fresh EVs or TGF‐*β*1 every two days (see Figure S2 in the Supporting Information). Throughout the experimental period, the activity of cells growing on the devices was monitored via daily OECT measurements. Figure 3a (see also Figure S3 in the Supporting Information) illustrates highly viable cells on the devices at the end of the experimental period, assessed by cytotoxicity/viability assays (LIVE/DEAD), revealing an exceedingly high ratio of live‐to‐dead cells, even after continuous TEV treatment. This also highlights the added benefit of using the OECT which has a transparent channel that can be used to image the cells. Cells present on the transistor channel and the gate electrode induce a shift in the cutoff frequency (Figure 3a,c), which decreases as the cells continue to grow and differentiate, creating a barrier to the ion flux and slowing the response of the device. This is equally reflected in the extracted cell layer resistance (*R*_cell_) data, which increase over time (Figure 3b,c and Figure S4 (Supporting Information)). A confluent layer of cells grown on the transistor channel induces a shift in the OECT cutoff frequency, from 860 Hz (dashed black line) to 140 Hz (solid blue line), and an increase in impedance from 4.9 to 26.4 kΩ (Figure 3b,c). The cutoff frequency was normalized to treatment day 0 for each condition (Figure 3d), as the treatments were started at this time and to account for device‐to‐device performance variation.

Statistical analyses were performed to analyze the effect of treatment condition and treatment time on cutoff frequency. Within conditions, a two‐way analysis of variance (ANOVA) (see the Experimental Section for reporting convention) showed that there was not a statistically significant difference in mean normalized cutoff frequency between day 0 and day 9 for nontreated cells (*F*(1,45) = 54.88, *p* = 0.92) with difference in means (*M*_diff_) = 0.17 (−17%) and cells treated with HEK–EVs (*F*(1,45) = 54.88, *p* = 0.86) with *M*_diff_ = 0.20 (−20%). A small decrease in normalized cutoff frequency is observed in both conditions, which may be caused by the tightening of the lateral cell–cell junctions, as cells continue to differentiate and form an insulating barrier on the device. An asymptotic regression analysis revealed that the normalized cutoff frequency of nontreated MCF10A cells tended toward 0.88 ± 0.034 (Figure S5a, Supporting Information).

**Figure 4:**
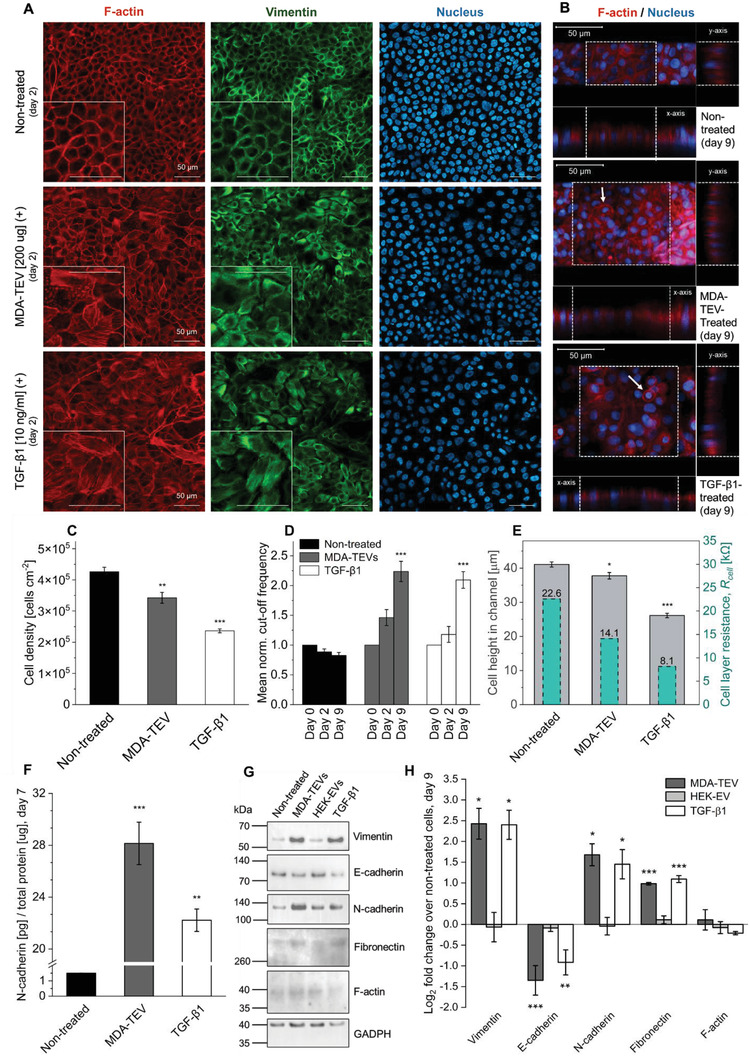
Biological validation of OECT‐based measurements to determine EMT status. a) Confocal images of MCF10A cells on treatment day 2. Cells were stained for F‐actin, vimentin, and nuclei. Insets show a close‐up of cells. Scale bars, 50 µm. b) Representative *x*/*z* and *y*/*z* orthogonal views (ortho‐views, right) of each cell layer in the OECT channel (treatment day 9), obtained by z‐stacked confocal images. PEDOT:PSS channel is indicated by white dotted lines. Cells were stained for F‐actin and nuclei. Scale bars, 50 µm. White arrows: cells with a rounded morphology. c) Cell density on treatment day 2 across conditions. Cell number was determined by counting cell nuclei from 5 frames (mean ± s.e.m.; *n* = 2). d) Normalized cutoff frequency from treatment day 0, 2, and 9 across conditions (mean ± s.e.m.; *n* = 3 and 2 for nontreated/MDA–TEV and TGF‐*β*1, respectively). e) Cell height in channel derived from orthogonal projections (mean ± s.e.m.; minimum 8 measurements taken from different *x*‐ and *y*‐positions; see Figure S8 and Videos S1–S3 in the Supporting Information) and the cell layer resistance measured in each specific channel as derived from impedance (*Z*) plots (*n* = 1). f) ELISA‐based N‐cadherin protein on treatment day 7 across conditions (mean ± s.e.m.; *n* = 1). g) Immunoblots of whole‐cell lysates collected on treatment day 9, probed with vimentin, E‐cadherin, N‐cadherin, fibronectin, F‐actin, and GADPH. Unedited blots are available in Figure S9 (Supporting Information). h) Quantitative values of protein expression derived from immunoblots presented as log_2_ fold‐change versus nontreated condition expression level (mean ± s.e.m.; *n* = 3). One‐way ANOVA: (c, e, and f); two‐way ANOVA: (d). * *p* ≤0.05, ** *p* ≤0.01, *** *p* ≤0.001.

There was, however, a statistically significant difference in mean normalized cutoff frequency between day 0 and day 9 for cells treated with MDA–TEVs (*F*(1,45) = 54.88, *p* < 0.001) with *M*_diff_ = 1.24 (+124%) and cells treated with TGF‐*β*1 (*F*(1,45) = 54.88, *p* < 0.001) with *M*_diff_ = 1.09 (+109%). This increase in cutoff frequency indicates an increase in the “leakiness” of the cell monolayer. This is in line with the degradation of cell–cell junctions,^[^ ^69^ ^]^ characteristic of cells undergoing EMT, leading to an increase in paracellular ion flux into the PEDOT:PSS channel.^[^ ^70^ ^]^ Optical imaging illustrates that cells are present and alive on the channel (Figures 3a and **4**b), and although the changes in paracellular ion flux cannot be resolved optically, the highly sensitive OECT‐based measurements reveal these changes in paracellular flux caused by EMT, enabling real‐time monitoring of cell phenotype transition. Interestingly, it appears that MDA–TEVs initially promote degradation of epithelial barrier integrity faster than TGF‐*β*1, during treatment days 0–4, after which point the effect of both treatments is comparable. This is in line with previously reported timescales of TEV and TGF‐*β*1‐indued EMT.^[^ ^50^, ^51^ ^]^

Additionally, mean normalized cutoff frequency was compared between treatment conditions on day 9 (Figure 3e). A two‐way ANOVA showed that there was a statistically significant difference in mean normalized cutoff frequency on day 9 between nontreated cells and cells treated with either MDA–TEVs (*F*(3,45) = 35.20, *p* < 0.001) with *M*_diff_ = 1.41 (171%) or TGF‐*β*1 (*F*(3,45) = 35.20, *p* < 0.001) with *M*_diff_ = 1.27 (153%). Whereas there was not a statistically significant difference in mean normalized cutoff frequency on day 9 between nontreated cells and cells treated with HEK–EVs (*F*(3,45) = 35.20, *p* = 1) with *M*_diff_ = 0.021 (−3%). This demonstrates our ability to compare different treatment conditions conducted on different OECT devices to one another, which holds promise for scaled‐up operation and confirms that the observed effect is caused by the intervention (MDA–TEV or TGF‐*β*1).

Finally, a simple linear regression was used to test if MDA–TEV treatment time significantly predicted the mean normalized cutoff frequency (Figure S6, Supporting Information). The fitted regression model was

(1) $$\begin{array}{ccc} & & \mathrm{Normalized}\;\mathrm{cut}-\mathrm{off}\;\mathrm{frequency}=1+\;0.134\;\left(\pm 0.008\right)\\ & & \;\times \;\mathrm{MDA}-\mathrm{TEV}\;\mathrm{treatment}\;\mathrm{time} \end{array}$$

The overall regression was statistically significant (*R* ^2^ = 0.978, *F*(1,69) = 1527.60, *p* < 0.001) and it was found that MDA–TEV treatment time significantly predicted mean normalized cutoff frequency (*β* = 0.134, *p* < 0.001). This indicates that the kinetics of the degradation of epithelial barrier integrity in this regime (0–9 days of MDA–TEV treatment) can be represented by a linear approximation (Equation (1)). We would assume that the linear regime only represents a slice of the epithelial barrier degradation process, as the cells progress through EMT. As cell barrier integrity invariably is associated with cell phenotype^[^ ^39^ ^]^ and by extension EMT status,^[^ ^1^, ^69^ ^]^ our platform is thus able to provide insight into the kinetics governing TEV‐mediated EMT. This is highly applicable to predicting therapeutic response and modeling the relationship between TEV/drug treatment dose/time and epithelial barrier integrity.

Compared to previous studies, which assessed the effects of MDA–TEVs up to 48 h post‐treatment,^[^ ^50^, ^51^ ^]^ we demonstrate continuous monitoring of MDA–TEV‐induced EMT over a 9 day period and establish a linear relationship between MDA–TEV treatment time and epithelial barrier integrity and thus EMT status.

## 4 Biological Validation of OECT Measurements by Immunofluorescence and Immunoblots

To validate the electrical readouts and establish cutoff frequency and impedance as a credible figure of merit for EMT, we assessed cell phenotype and determined EMT status by analysis and quantification of gene expression of classic EMT markers using immunoblots and enzyme‐linked immunosorbent assay (ELISA). Additionally, we performed immunofluorescence (IF) confocal imaging to assess protein abundance and location profiles and to measure cell height and assess morphology. IF confocal imaging was performed both on samples fixed 48 h after treatment, as previous studies report TEV‐induced changes within this timeframe,^[^ ^50^, ^51^ ^]^ and on samples fixed on treatment day 9. A N‐cadherin‐based ELISA was performed on treatment day 7 and whole‐cell lysates were collected on treatment day 9 for immunoblot analysis.

After 48 h of exposure to MDA–TEVs or TGF‐*β*1, vimentin expression markedly increased in the treated cells and remodeling of F‐actin is apparent with the appearance of actin stress fibers inside the cells, as opposed to the cortical thin actin bundles in the nontreated cells, signifying focal adhesion maturation (Figure 4a and Discussion S3 (Supporting Information)). Additional confocal IF images of MCF10A cells stained for E‐cadherin, N‐cadherin, and nuclei are available in Figure S7 (Supporting Information). After 9 days of treatment, F‐actin stress fibers remained present (Figure 4b). Cell density, as measured by the number of nuclei per area, decreased by 23% and 44% with MDA–TEV and TGF‐*β*1 treatment, respectively (Figure 4c). This is caused by individual cells occupying a larger area, which indicates a flattening of the cells, consistent with the transformation from the apical–basal polarity of epithelial cells to the back–front polarity of migratory mesenchymal cells.^[^ ^69^ ^]^ Cells with a rounded morphology were also observed in the transistor channel (Figure 4b). The accompanying functional changes to the cells was captured by the OECT measurements, as cutoff frequency increased in the same 48 h period (Figure 4d), indicating that changes to lateral cell–cell adhesion have already begun and is readily detectable by the OECT.

To further substantiate these findings and demonstrate the multiparametric readout capabilities of the OECT platform, confocal microscopy was employed to evaluate the height of cells hosted on the OECT channel, as changes in cell height can be used to imply phenotypic switching during EMT.^[^ ^71^ ^]^ Z‐stacked confocal images of cells in the OECT channel, labeled for F‐actin and nuclei, were obtained on day 9 of the experimental period (Figure 4b and Figure S8 and Videos S1–S3 (Supporting Information)). Cell height varied between nontreated and treated cells, mirroring the trend of cell layer resistance derived by OECT monitoring (Figure 4e), and clearly demonstrating the unique ability of our platform to directly correlate a biological event with a corresponding electrical readout, both occurring in the channel. To assess the degradation of cell–cell junctions and determine EMT status, the samples were probed for several hallmark EMT proteins. MDA–TEV and TGF‐*β*1 treatment increased the abundance of vimentin, N‐cadherin, and fibronectin (mesenchymal markers), and decreased the abundance of E‐cadherin (epithelial marker), while F‐actin remained constant (Figure 4f–h and Figure S9 (Supporting Information)). This evidences the occurrence of the cadherin switch (from E‐ to N‐cadherin) and corroborates vimentin upregulation, as seen on the confocal images (Figure 4a). ELISA‐based N‐cadherin protein quantification revealed that N‐cadherin on treatment day 7 in the MDA–TEV‐ and TGF‐*β*1‐treated cells was 19‐fold and 15‐fold higher, respectively, compared to the nontreated cells (Figure 4f). TWIST1, a marker of EMT, was also more abundant in MDA–TEV‐treated cells (Figures S10 and S11, Supporting Information). Cells treated with HEK–EVs did not exhibit differential abundance of these proteins (Figure 4g,h), indicating that it is specifically MDA–TEVs that mediate EMT. Collectively, these results strongly indicate that MDA–TEV treatment induces EMT in MCF10A cells.

## 5 OECT‐Based Screening Reveals That Heparin Treatment Prevents TEV‐Induced EMT

EMT represents an attractive target in oncology, however, direct targeting of EMT effector molecules is, in most cases, pharmacologically challenging.^[^ ^72^ ^]^ Previous anti‐EMT strategies have targeted signaling pathways, molecular drivers, or the mesenchymal cells themselves,^[^ ^73^ ^]^ and several clinical trials targeting EMT are ongoing.^[^ ^74^ ^]^ Recent efforts using nitrogen and its analogs to interfere with the process of EMT have proven efficacious in blocking TNBC metastasis and invasiveness both in vivo and in vitro.^[^ ^75^ ^]^ Such reports underscore the importance of identifying pharmacological targets against EMT to prevent metastasis. TEVs are one such target and attenuating TEV uptake as a therapeutic intervention is a strategy.^[^ ^53^, ^59^, ^76^ ^]^

**Figure 5:**
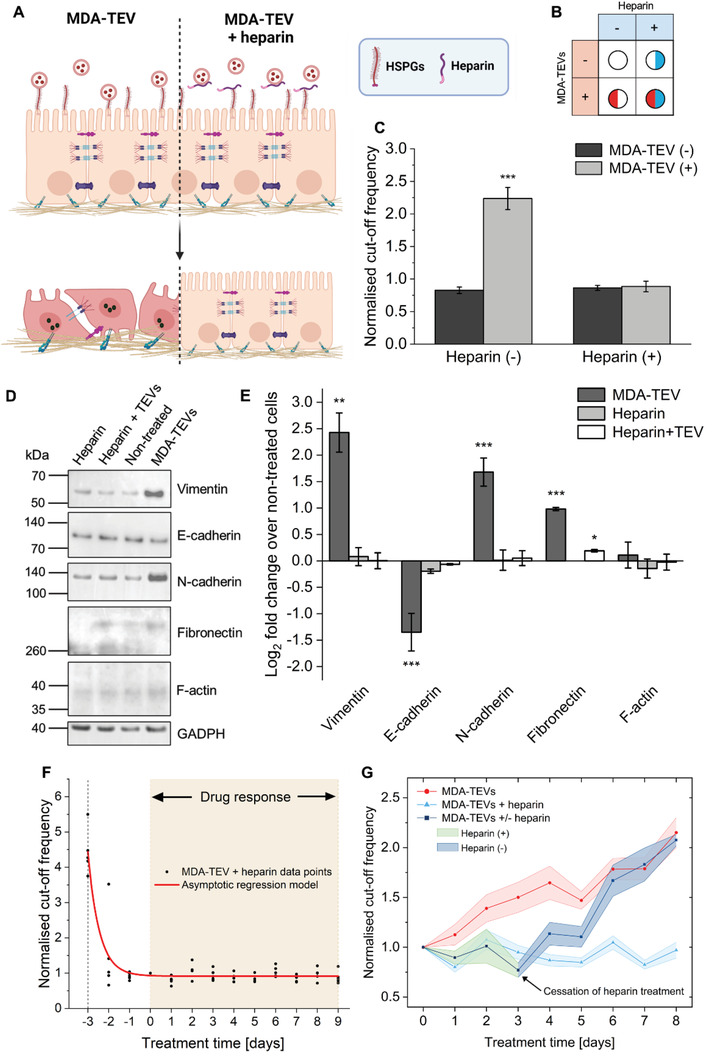
OECT‐based phenotypic screening of heparin as a TEV‐targeting, anti‐EMT drug. a) Schematic showing the proposed mechanism by which heparin binds to spike glycoproteins to outcompete cell surface HSGPs and prevent TEV adhesion and uptake. b) Two‐factor factorial design to assess the effect of heparin treatment on MDA–EV‐induced EMT. c) Comparison of the normalized cutoff frequency on treatment day 9 between cells treated with MDA–TEVs in the absence (−) or presence (+) of heparin (mean ± s.e.m.; *n* = 3). d) Immunoblots of whole‐cell lysates collected at the end of the experiment. Equal quantities of protein were separated on SDS‐PAGE gels and membranes were blotted with indicated antibodies (same antibodies as listed in Figure 4). Unedited immunoblots are available in Figure S9 (Supporting Information). e) Quantitative values of protein expression derived from immunoblots presented as log_2_ fold‐change versus nontreated expression level (mean ± s.e.m.; *n* = 3). f) Asymptotic regression model fitted to the drug response cutoff frequency data points. g) Cells were treated with heparin between days 0 and 3 (green colored area) while being exposed to MDA–TEVs. Washing with PBS and subsequently exposing cells to MDA–TEVs lead to an increase in cutoff frequency (dark blue colored area). Data points before treatment day 0 are omitted for clarity (mean ± s.e.m.; s.e.m. indicated by lightly colored areas; *n* = 2 and 3 for the transient heparin treatment condition (dark blue data points) and MDA–TEV (+/−) heparin (red and light blue data points), respectively). Two‐way ANOVA. * *p* ≤0.05, ** *p* ≤0.01, *** *p* ≤0.001.

We selected heparin as a possible TEV‐targeting, anti‐EMT treatment and leveraged our platform to screen this candidate drug. Heparin is a glycosaminoglycan commonly used as an anticoagulant drug. However, it is reported to have anticancer effects in humans^[^ ^77^, ^78^, ^79^ ^]^ and has been shown to decrease metastasis in animal models.^[^ ^80^ ^]^ The mechanism for heparin's antimetastatic effect has been proposed to be a blocking function in tumor cell/platelet interactions.^[^ ^81^, ^82^ ^]^ In addition to this mechanism, heparin's anticancer role may involve regulating TEV uptake into recipient cells.^[^ ^83^ ^]^ EVs depend on cell‐surface heparan sulfate proteoglycans (HSPGs) for their internalization and functional activity, and heparin is a competitive inhibitor of cell surface receptors dependent on HSPG coreceptors.^[^ ^53^, ^76^, ^83^ ^]^ Heparin treatment of cells and/or EVs is known to interfere with TEV binding to the cell surface^[^ ^53^, ^76^, ^83^ ^]^ (**Figure** **5**a), and heparin has been reported to partially block EV transfer of epidermal growth factor receptor variant III (EGFRvIII) mRNA into recipient cells^[^ ^83^ ^]^ and significantly reduce EV‐mediated stimulation of cancer cell migration and invasion.^[^ ^52^, ^53^ ^]^ This function makes HSPGs a potential target for inhibition of TEV‐mediated tumor invasion and metastasis. To test the efficacy of heparin in preventing MDA–TEV‐induced EMT, a two‐factor factorial experiment was designed, where cells were incubated with or without MDA–TEVs in the presence or absence of heparin (Figure 5b).

Heparin treatment of cells effectively ablated the EMT‐promoting effect of MDA–TEVs and heparin itself does not appear to adversely affect the barrier forming properties of the MCF10A cells (Figure 5c and Figure S12 (Supporting Information)), the abundance of several mesenchymal and epithelial markers (Figure 5d–f), nor the viability of cells (Figure S3, Supporting Information). We hypothesize that if heparin treatment blocks MDA–TEV binding to cells and thereby prevents MDA–TEV‐induced EMT, then it may have an impact on cancer metastasis and could therefore be a starting point in developing an effective new drug for treating metastatic TNBC.

To demonstrate the facile drug response modeling enabled by the OECT time‐series data, an asymptotic regression was used to test if heparin treatment time (concurrent with MDA–TEV exposure) significantly predicted the normalized cutoff frequency (Figure 5f). The fitted regression model was

(2) $$\begin{array}{ccc} & & \mathrm{Normalized}\;\mathrm{cut}-\mathrm{off}\;\mathrm{frequency}\\ & & \;=\;a\;-\;\beta \;\times \;\gamma^{\left(\mathrm{MDA}-\mathrm{TEV}\;\mathrm{treatment}\;\mathrm{time}\right)} \end{array}$$

The overall regression was statistically significant (*R* ^2^ = 0.872, *F*(2,62) = 210.63, *p* < 0.001) and it was found that heparin treatment time significantly predicted normalized cutoff frequency (*α* = 0.92, *β* = −3.52, *γ* = 0.16, *p* = 0.0013), which compares well with the same model applied to the nontreated condition (Figure S6, Supporting Information). This demonstrates our ability to readily model the transient drug response (Equation (2)) and compare it to a control condition, a key feature for comparing therapeutic efficacy when screening several drug candidates.

It has been reported that persistent heparin treatment is necessary to abrogate the malignant effects of TEVs and inhibit TEV‐induced tumor progression.^[^ ^84^ ^]^ To test the transient effect of heparin treatment and demonstrate the capability of our platform, cells were concurrently treated with heparin and MDA–TEVs for 3 days, after which the cells were washed in phosphate‐buffered saline (PBS) and heparin treatment was ceased while MDA–TEV treatment continued (Figure 5g). Our results confirm the transient effect of heparin treatment and demonstrate that persistent treatment of cells with heparin concurrently with exposure to MDA–TEVs is necessary to prevent MDA–TEV‐induced EMT. Crucially, this also demonstrates the ability of the OECT to continuously monitor a dynamic drug response and investigate the temporal relationship noninvasively with a quantitative readout and in a facile manner.

## 6 Discussion

Interventions that target EMT drivers may be useful therapeutic strategies against cancer metastasis. A few successful clinical trials in human patients using EMT‐targeting drugs provide proof of principle that such interventions have translational implications.^[^ ^74^ ^]^ However, there are limitations associated with these existing drugs (such as lack of specificity and adverse effects) and a shortage of phenotypic screening technologies by which to rapidly identify new and more effective molecular entities with high potential for translation to in vivo outcomes. Here, we report the development of a new phenotypic screening platform based on bioelectronic technology for the real‐time monitoring of TEV‐induced EMT and testing of antimetastatic drugs. Current phenotypic screening technologies suffer from limitations that hitherto have frustrated adoption for preclinical development,^[^ ^17^ ^]^ where the choice is often between dynamic monitoring or high throughput. By contrast, our platform offers real‐time, multiparametric monitoring of a pathogenic process in vitro and has a direct path toward scaling and massive parallelization. By integrating OECTs with a disease‐relevant model of TEV‐driven invasive ductal carcinoma, we achieved real‐time, label‐free, noninvasive monitoring of MDA–TEV‐induced EMT over a 9 day period, almost fivefold longer than previous reports.^[^ ^50^, ^51^ ^]^ Through validation of the electrical signal with on‐chip and end‐point biomolecular assays, we established OECT‐based electrical measurements as a novel figure of merit for determining EMT status. To demonstrate its preclinical utility, we used our platform to identify heparin as a candidate antimetastatic drug that acts by blocking TEV function to prevent EMT. These data provide proof of concept that TEV blocking compounds can be identified with OECT‐based functional phenotypic screens targeting EMT and confer benefit in the treatment of TEV‐mediated, metastasis‐initiating EMT. Future work investigating the use of other general inhibitors^[^ ^85^ ^]^ or specific inhibitors,^[^ ^59^, ^86^ ^]^ e.g., antibodies, would be of great interest to provide additional proof of concept data for the platform. Furthermore, our platform can be used to make drug repurposing predictions and indicate potential for further clinical evaluation, as is the case with heparin. Repurposing of FDA‐approved medications entails a lower risk of failure, accelerates the time frame for drug development, and sees substantial savings in preclinical and phase I and II costs;^[^ ^87^ ^]^ a potential avenue for addressing the current costly state of drug development. As such, our platform adds to the toolbox of translational, preclinical assays.

TEVs represent a novel and potentially more specific target for antimetastasis therapies. By using our platform, we can explore this new therapeutic window between cancer and normal cells with continuous, multiparametric readouts. The compatibility of our platform with on‐chip optical imaging and end‐point assays for multiparametric readouts is crucial for establishing OECT‐based measurements as a functional and robust figure of merit for EMT status. This feature is necessary for validation and qualification of analytical performance and biological in vivo relevance, and for adoption by industry and regulatory agencies.^[^ ^21^ ^]^ Further, our platform facilitates quantitative translation to in vivo outcomes, as it readily allows modeling of pharmacokinetic/pharmacodynamic relationships, crucial for elucidating the causative relationship between drug exposure and response.^[^ ^88^ ^]^ Although our platform improves upon current phenotypic screening technologies, positive hits must still be screened in animal models prior to clinical trials. Here, the facile drug response modeling enabled by the platform may help inform appropriate dosage and dose timings, reducing the reliance on animal studies in line with the three Rs principle for more ethical animal testing. Additionally, by integrating patient‐derived samples with our platform, we may decrease the risk of failure, especially due to lack of efficacy, at the clinical phase.

Functional, electrical monitoring represents a new tool for elucidating the role of TEVs in EMT that readily facilitates extraction of the kinetics governing TEV‐driven healthy epithelial barrier disruption. Such readouts have the potential to implicate specific EV subtypes, reveal TEV dose–response relationships, and explore the function of EVs derived from cancerous/noncancerous cell lines and clinical samples. Finally, our platform is amenable to integration with models recapitulating other pathologies where epithelial barrier function serves as a functional readout of pathogenesis/disease progression.

In summary, the functional, bioelectronic phenotypic screening platform described here demonstrates the feasibility to monitor TEV‐induced EMT in real time and rapidly identify potent compounds that act on TEV function and are effective in the treatment of metastasis‐initiating EMT in preclinical models. This platform will facilitate the efforts to target TEV‐driven EMT and aid the development of first‐in‐class antimetastatic drugs.

## 7 Experimental Section

### Study Design

The objectives of this study were i) to develop a phenotypic screening platform based on bioelectronic technology to dynamically monitor TEV‐induced EMT, ii) to validate the electrical readouts with biomolecular assays, and iii) to test the blocking effect of heparin on TEV function and provide proof of principle that OECT phenotypic screening can identify potential antimetastatic drug candidates. The effects of different treatments on MCF10A phenotype and EMT status were assessed in a controlled laboratory experiment using continuous OECT‐based measurements and a range of in vitro assays. The treatments included exposure to EVs derived from MDA‐MB‐231 and HEK‐293 cells (from more than four different cell passages), TGF‐*β*1, and heparin. MDA–TEV, nontreated, heparin, and heparin + MDA–TEV experiments were performed 3 times and TGF‐*β*1, HEK–EV, and transient heparin experiments were performed twice with different MCF10A cell passages. Experiments were performed on both OECTs and in cell culture plates in parallel and stopped at prospectively defined study endpoints.

### Cell Culture

MDA‐MB‐231 epithelial breast cancer cells (ATCC HTB‐26) were cultured according to supplier's instructions with Dulbecco's Modified Eagle Medium (DMEM; Thermo Fisher Scientific, TFS) and supplemented with 10% v/v fetal bovine serum (FBS; Merck), 50 U mL^−1^ penicillin, 50 µg mL^−1^ streptomycin, and 1% v/v GlutaMAX (TFS). MDA‐MB‐231 (ATCC HTB‐26) cells were cultured to 80% confluency before passaging and did not exceed 15 passages. HEK‐293 cells (kind gift from Marc Borsotto, Université de Nice Sophia Antipolis, France) were cultured with Advanced DMEM (TFS) and supplemented with 10% v/v FBS (Merck), 50 U mL^−1^ penicillin and 50 µg mL^−1^ streptomycin (TFS), 1% v/v GlutaMax (TFS), and 50 µg mL^−1^ gentamicin (TFS). HEK‐293 cells were cultured to 70–80% confluency before passaging and did not exceed 15 passages. MCF10A human mammary epithelial cells (ATCC CRL‐10317) were cultured with Mammary Epithelial Cell Growth Medium Basal Medium (CC‐3151; Lonza) and supplemented with growth supplements bovine pituitary extract (BPE), 2 mL; human epidermal growth factor (hEGF), 0.5 mL; hydrocortisone, 0.5 mL; and insulin, 0.5 mL from MEGM SingleQuots Supplement Pack (CC‐4136; Lonza) and 100 ng mL^−1^ cholera toxin (Sigma‐Aldrich). MCF10A were cultured to 70–80% confluency before passaging and did not exceed 10 passages/trypsin neutralizing solution was used during subculturing. All cell lines were incubated at 37 °C with 5% CO_2_.

### EV Isolation, Characterization, and Western Blot Analysis—Extracellular Vesicle Isolation

For EV harvesting, MDA‐MB‐231 and HEK‐293 cells were cultured until 80% confluent, washed twice with PBS (TFS), and incubated with serum‐free culture medium for 48 h before the conditioned medium (CM) was collected for further processing. CM from MDA‐MB‐231 and HEK‐293 cells was processed using an adapted protocol.^[^ ^89^ ^]^ CM was centrifuged at 4 °C, 3000 × *g* for 30 min to remove cells and cellular debris. The supernatant was centrifuged at 4 °C, 100 000 × *g* for 4 h (Type 50.2 Ti Fixed‐Angle Rotor, Beckmann XL‐90 Ultracentrifuge) to pellet out EVs. The pellet was resuspended in 500 µL MCF10A cell culture medium or PBS (depending on the downstream application) and stored at 4 °C. For cell experiments, EVs were used within 2 days of isolation, and for particle imaging (TEM) and size analysis, EVs were analyzed on the same day.

### EV Isolation, Characterization, and Western Blot Analysis—TEM

EV samples were visualized by TEM using negative staining method. 10 µL sample was applied on glow‐discharged carbon‐coated copper grids for 2 min, washed twice in Milli‐Q water for 30 s each, and stained with 1% w/v water solution of uranyl acetate for 2 min at room temperature (RT). Imaging was performed on a FEI Tecnai G20 electron microscope, operating at 200 kV, 20 µm objective aperture, and images were recorded with a camera (AMT).

### EV Isolation, Characterization, and Western Blot Analysis—Immunogold Staining

TEVs from MDA‐MB‐231 cells were incubated with mouse anti‐CD63 (TFS, 1:5000) followed by anti‐mouse conjugated with 20 nm gold nanoparticle (1: 5000; TFS) for 2 h. TEVs were spun through a G25 column (Cytiva) after each incubation step and imaged using TEM.

### EV Isolation, Characterization, and Western Blot Analysis—EV Size, Concentration, and Zeta Potential Characterization

NTA was used to determine the size and particle concentration of EVs. Samples were diluted 100‐fold in PBS and tracked on a Nanosight NS500 (Malvern). Zeta potential was determined using a folded capillary zeta cell (DTS1070, Malvern) and a Zetasizer Nano ZSP (Malvern). Samples were dispersed in PBS (pH 7.4) and the Smoluchowski model was applied. The total protein content of intact EVs was quantified using a Qubit 4 Fluorometer (TFS) as per manufacturer's instruction.

### EV Isolation, Characterization, and Western Blot Analysis—Cell Lysate and EV Sample Protein Extraction

Cultured cells were washed twice with cold PBS and solubilized in 1 mL radio immunoprecipitation assay buffer (RIPA) Lysis and Extraction Buffer supplemented with 10 µL Halt Protease Inhibitor Cocktail and 10 µL ethylenediaminetetraacetic acid (TFS) immediately before use. Samples were incubated on ice for 5 min with agitation (20–30 min for MCF10A cells with scraping), aspirated buffer was collected into a microcentrifuge tube and centrifuged at 4 ^○^C; 14 000 × *g* for 15 min to pellet cell debris. The supernatant (cell lysate) was collected and stored at −80 °C pending further analysis. EV protein samples were prepared by resuspending pellets post‐ultracentrifugation in the supplemented RIPA buffer.

### EV Isolation, Characterization, and Western Blot Analysis—Western Blot

The total protein content of cell lysates and EV samples was quantified using a Qubit 4 Fluorometer (TFS) as per manufacturer's instruction. Samples were diluted in lithium dodecyl sulfate sample buffer (4X Bolt; Invitrogen), denatured by heating for 10 min at 70 °C, and subjected to electrophoresis using precast NuPAGE Novex 4–12% Bis‐Tris Proteins Gels (Invitrogen) in NuPAGE 2‐(N‐morpholino)ethanesulfonic acid (MES) sodium dodecyl sulfate (SDS) Running Buffer (Invitrogen) under nonreducing conditions at constant 200 V for 30 min. The same amount of protein (between 10–25 µg) from each sample was loaded into the gel wells. Proteins were electrotransferred onto polyvinylidene fluoride (PVDF) Transfer Membranes (TFS) using NuPAGE Transfer Buffer (Invitrogen). Membranes were blocked with 5% w/v bovine serum albumin (BSA) in PBS (TFS, Oxoid) with 0.1% v/v Tween‐20 (phosphate buffered saline with tween (PBST); TFS) for 1 h. For EV blots, membranes were probed with mouse anti‐CD9 (Invitrogen; 1:5000), mouse anti‐CD63 (TFS, 1:5000), mouse anti‐annexin A1 (TFS; 1:5000), and rabbit anti‐calreticulin (abcam; 1:5000) monoclonal primary antibodies for 1 h in PBST. For MCF10A blots, membranes were probed with mouse anti‐vimentin (abcam; 1:1000), mouse anti‐F‐actin (abcam, 1:500), rabbit anti‐E‐cadherin (abcam; 1:1000), mouse anti‐N‐cadherin (1:1000), mouse anti‐TWIST1 (1:250), and mouse anti‐GADPH (Sigma‐Aldrich; 1:25 000) monoclonal primary antibodies for 1 h in PBST. This was followed by incubation with HRP‐conjugated anti‐mouse IgG H&L (Invitrogen; 1:10 000) and anti‐rabbit IgG H&L (abcam; 1:10 000) secondary antibodies in PBST for 1 h. All incubations were carried out on a shaker at RT or 4 °C. Western blots were washed 3–5 times in PBST for 5 min after each incubation step and were visualized using Pierce enhanced chemiluminescence Western Blotting Substrate (TFS) on a G:BOX Chemi XX6 (Syngene). Band intensity was quantified using ImageJ Software Band function and normalized with GADPH to compare relative protein expression between conditions.

### ELISA

To investigate the expression of mesenchymal marker N‐cadherin in MCF10A cells before and after treatment with MDA–TEVs, an ELISA assay was done. Following the manufacturer's protocol, a Human N‐cadherin DuoSet ELISA kit (R&D Systems) was utilized to measure the concentration of N‐cadherin in MCF10A lysates on day 7 of treatment. Absorbance at 450 nm, as recommended, was measured for all samples, and the concentration of N‐cadherin was found using the absorbance and concentration of the protein standards and the absorbance of the unknown concentration samples. The concentration of N‐cadherin protein present in MCF10A lysates was normalized by total protein content.

### Cell‐Based EMT Experiments on OECTs

OECTs were cleaned thoroughly with Milli‐Q water and isopropanol prior to sterilizing by immersion in 70% ethanol for 24 h. The devices were left in a sterile microbiological safety cabinet (MSC) to promote evaporation of ethanol before incubating with normal culture medium overnight. Cloning cylinders with an area of 0.5 cm^2^ doubled as culture wells which were stuck onto OECT devices with PDMS. The wells defined the area in which the cells were cultured, with 3–6 channels and a gate electrode accessible within each well. MCF10A cells were then seeded at 1 × 10^4^ cells cm^−2^ and cultured for 3 days until cell coverage was ≈70–80%, covering all channels fully, at which point the cells displayed a polygonal morphology, characteristic of an epithelial phenotype (Figure 3a). MCF10A cells were usually serum‐starved for EMT studies^[^ ^90^ ^]^ but the normal culture medium did not contain serum, therefore, the base medium was kept the same for all conditions. Treatment was started on culture day 3 (called treatment day 0) and cell culture medium either presupplemented with 200 µg MDA–TEVs, 185 µg HEK–EVs, 10 ng mL^−1^ TGF‐*β*1, heparin, or without supplementation. The discrepancy in dose between MDA–TEVs and HEK–EVs was due to the slight difference in protein‐to‐vesicle ratio of each EV type (see Table 1). The total protein content of EV preparations was used as a proxy for particle concentration. EV dose was normalized to the number of MCF10A cells seeded to ensure that an equal number of cells were exposed to an equal number of EVs (in this case 12 × 10^9^ vesicles per 5000 cells seeded), regardless of culture area and medium volume. This meant that an equal number of MCF10A cells were exposed to an equal number of EV particles, both TEVs and HEK–EVs. EV doses of 50–200 µg were typical^[^ ^51^, ^91^, ^92^ ^]^ and a 5 ng mL^−1^ dose of TGF‐*β*1 induced EMT in MCF10A cells,^[^ ^90^ ^]^ but prolonged treatment with 5 ng mL^−1^ TGF‐*β*1 (6 days) was necessary to induce EMT in MCF10A cells,^[^ ^93^ ^]^ therefore the dose was increased to 10 ng mL^−1[^ ^94^ ^]^ to accelerate this process. Supplemented exogenous EVs were isolated from their respective cell lines every 2 days and used fresh to prevent degradation of EV function by prolonged storage times. It was important to acknowledge that the dose of EVs used for in vitro studies typical exceeded those found in vivo. However, loss of function during harvesting, isolation, and storage required a higher number of EVs to be applied to ensure that a critical number of functional EVs interacted with the cells. Electrical measurements were performed prior to medium changes. Cells in the OECTs were fixed for IF staining, lysed to collect whole‐cell lysates for immunoblot analysis, or used to assess cell viability on treatment day 9 (after 12 days of culture).

### Heparin Treatment

Cells were incubated with or without 200 µg MDA–TEVs in the presence or absence of 10 µg mL^−1[^ ^52^, ^53^, ^83^ ^]^ heparin sodium salt from porcine intestinal mucosa (Sigma‐Aldrich). Heparin treatment was refreshed every 48 h concurrently with medium changes (supplemented with MDA–TEVs or not). In the transient heparin treatment study, cells were treated with MDA–TEVs and heparin concurrently until day 3, after which time the cells were washed thoroughly with PBS 3 times^[^ ^83^ ^]^ and treated with MDA–TEVs for the remainder of the experiment.

### Fabrication of OECTs

OECTs were microfabricated on glass substrates based on established protocols using standard photolithography and parylene C peel‐off techniques.^[^ ^95^, ^96^ ^]^ The process started with the first layer of photoresist (AZ5214) being spin‐coated and exposed to ultraviolet light using contact aligner to create Au electrodes and interconnection pads. The photoresist patterns were generated with AZ 726 MIF developer, followed by metal sputtering of 10 nm Cr and 100 nm Au and a standard lift‐off process using hot dimethyl sulfoxide. Next, the second layer of photoresist AZ9260 was coated on the substrates and developed using AZ developer. A parylene C layer was deposited to insulate the gold interconnects. The OECT channel and gate were patterned by reactive ion etching, using a second layer of parylene C, which was peeled off to yield a channel length and width of 50 or 100 µm and a gate dimension of 500 × 500 µm^2^.

### Organic Electrochemical Transistor Measurements

OECTs were characterized using a dual‐channel source‐meter unit (NI‐PXI) with custom‐written control code in LabVIEW. All measurements were performed using the integrated planar gate electrodes. The channels studied were either 50 × 50 or 100 × 100 µm in length and width.

### Extraction of Cell Layer Resistance from Z versus Frequency Plots

The electrochemical impedance spectra (EIS) was obtained from the OECT channels using the source, drain, and gate as the three electrode setup and a custom made MATLAB code as described earlier by Rivnay et al.^[^ ^67^ ^]^ EIS was performed at a frequency range between 100 kHz to 0.1 Hz at *V* = 0 V versus *V*_OC_ with an AC amplitude of 10 mV. The resistance value for each condition was extracted by using Equation (3) and the impedance magnitude value at 2 kHz, assuming that capacitance contribution to the overall signal was negligible

(3) $$Z\;=\;\sqrt{R^{2}\;+\;\frac{1}{\omega^{2}C^{2}}}$$

### OECT Data Analysis

Cut‐off frequency (CF) was normalized to treatment start date using the following equation: normalized CF = CF_*n*_/CF_*n* = 0_, where *n* is the treatment day and CF_*n* = 0_ refers to the cutoff frequency on treatment day 0.

### Confocal Microscopy—Viability Assay

The viability of cells growing on the OECT platform was checked by evaluating plasma membrane intactness and enzymatic activity via a two‐color fluorescence cytotoxicity/viability assay (LIVE/DEAD Viability/Cytotoxicity Kit, for mammalian cells, Invitrogen). The assay was performed at the end of the experimental period (treatment day 9; culture day 12), by removing cell growth medium, washing thoroughly with PBS and adding the reagents. After 1 h incubation, the reagent was replaced with PBS before visualization with a confocal microscope.

### Confocal Microscopy—Immunofluorescence Staining

All samples were fixed with 4% paraformaldehyde (TFS) for 10 min, at room temperature; then, samples were thoroughly washed with PBS and stored at 4 °C until ready to use. Prior to immunofluorescently labeling the samples, cells were permeabilized in 0.1% v/v Triton X‐100 (Fisher) for 10 min and then blocked for nonspecific binding with 1% w/v BSA (TFS) and 0.1% v/v Tween‐20 (TFS) in PBS for 1 h at room temperature. The primary and secondary antibodies used were Phalloidin‐iFluor 594 Reagent (abcam), Bisbenzamide H (Hoechst 33342) (abcam). Images were obtained using an epifluorescence/confocal microscope (Axio Observer Z1 LSM 800, Zeiss), using 10×/0.45 and 20×/0.8, (Plan‐Apochromat, Zeiss) objective. The 2D microscopy images shown were representative of three different frames/locations of each.

### Confocal Microscopy—Z‐Stacks and Cell Height

For each condition/treatment, z‐stacked confocal images were acquired from at least one channel of the respective OECT devices (independent samples). After acquiring these, the height of cells on the OECT channel was measured using the ortho‐view (*x*/*z*‐ and *y*/*z*‐planes) and graphic features/tools of the confocal microscope software (Axio Observer Z1 LSM 800, Zeiss; ZEN blue edition 3.4). The mean cell height for each condition/channel/sample was calculated from at least eight measurements: the *z*‐plane was fixed in the middle/center of each cell layer on the respective OECT channel, as identified by the z‐stack series data, and *x*‐ and *y*‐locations were varied within this frame to account for variations of the cell layer height on this surface/plane.

### Cell Density from IF Microscopy Images

IF images were analyzed using ImageJ software. Cell count was performed using the software. Images were converted to 8‐bit, and the threshold was adjusted to minimize noise and amplify contrast. The binary tools Fill Holes and Watershed were used to automatically determine cell borders. Cells were automatically counted using the Analyze Particles tool. Both cell counts including and excluding image borders were performed, and their mean was used as the final cell count.

### Statistical Analysis

For all experiments performed in this study, variance analysis was performed using a one‐way or two‐way ANOVA, depending on the number of factors, to find significant differences between conditions. *F*‐ and *p*‐values were reported using the following convention: *F*(df regression, df (degrees of freedom) residual) = [*F*‐value], *p* = [*p*‐value]). *M*_diff_ was the difference in means between the two conditions (listed in absolute terms and as a percentage). All data were plotted and analyzed using Microsoft Excel and OriginLab Pro and they were presented as mean ± standard error of the mean (s.e.m.). Statistical significance levels were determined as follows: *p ≤ 0.05, **p ≤ 0.01, and ***p ≤ 0.001. Each independent experiment corresponded to a biological replicate where cells from different passages were used; *n* = total number of biological replicates. Primary data are reported in Data File S1 (Supporting Information).

## Conflict of Interest

The authors declare no conflict of interest.

## Author Contributions

W.C.T., J.U., A.H., M.H. designed and performed the experiments. V.D. fabricated the OECTs. W.C.T., J.U., C.‐M.M., D.H., A.S., Z.L., A.‐M.P. analyzed the data. V.D., A.H., A.K. developed OECT measurements. W.C.T., J.U., C.‐M.M., M.H., A.M.R.G. performed biomolecular analytical assays. W.C.T., R.M. characterized EVs. D.H., S.P., R.F. extracted DNA and performed methylation qPCRpcr. W.C.T., A.‐M.P., S.I., S.D., R.M.O. conceptualized the study and designed experiments. W.C.T., A.‐M.P., R.M.O. wrote the paper.

## Supporting information

Supporting Information

Supplemental Video 1

Supplemental Video 2

Supplemental Video 3

Supplemental Table 1

## Data Availability

The data that support the findings of this study are available in the supplementary material of this article.
