# Supplementary material for: Organic Electronic Platform for Real‐Time Phenotypic Screening of Extracellular‐Vesicle‐Driven Breast Cancer Metastasis
Source: Adv Healthc Mater. 2023 May 21;12(27):2301194. doi: 10.1002/adhm.202301194 (PMC11468090; doi:10.1002/adhm.202301194)
Supplement: Supplementary file 1 — Supporting Information [file ADHM-12-2301194-s001.pdf]

ADVANCED HEALTHCARE MATERIALS

Supporting Information

for *Adv. Healthcare Mater.*, DOI 10.1002/adhm.202301194

Organic Electronic Platform for Real-Time Phenotypic Screening of Extracellular-Vesicle-Driven Breast Cancer Metastasis

*Walther C. Traberg, Johana Uribe, Victor Druet, Adel Hama, Chrysanthi-Maria Moysidou, Miriam Huerta, Reece McCoy, Daniel Hayward, Achilleas Savva, Amaury M. R. Genovese, Suraj Pavagada, Zixuan Lu, Anil Koklu, Anna-Maria Pappa, Rebecca Fitzgerald, Sahika Inal, Susan Daniel and Róisín M. Owens**

Supporting File

Organic electronic platform for real-time phenotypic screening of extracellular vesicle-driven breast cancer metastasis

Walther. C. Traberg[1], Johana Uribe[2], Victor Druet[3], Adel Hama[3], Chrysanthi-Maria Moysidou[1], Miriam Huerta[2], Reece McCoy[1], Daniel Hayward[4], Achilleas Savva[1], Amaury M. R. Genovese[1], Suraj Pavagada[1,4], Zixuan Lu[1], Anil, Koklu[3], Anna-Maria Pappa[1,5,6], Rebecca Fitzgerald[4], Sahika Inal[3], Susan Daniel[2], Róisín M. Owens[1]

[1]Department of Chemical Engineering and Biotechnology, University of Cambridge, Cambridge CB3 0AS, United Kingdom

[2]Robert F. Smith School of Chemical and Biomolecular Engineering, Cornell University, Olin Hall, Ithaca, New York 14853, United States.

[3]Biological and Environmental Sciences and Engineering Division, King Abdullah University of Science and Technology (KAUST), Thuwal 3955, Kingdom of Saudi Arabia

[4]Early Cancer Institute, University of Cambridge, Hutchison Research Centre, Cambridge, CB2 0XZ, United Kingdom

[5]Healthcare Innovation Engineering Center, Khalifa University, Abu Dhabi, United Arab Emirates

[6]Department of Biomedical Engineering, Khalifa University of Science and Technology, PO BOX 127788, Abu Dhabi, United Arab Emirates

Table of Contents

METHODS AND MATERIALS 3

SUPPLEMENTARY FIGURES

Supplementary Fig. 1: Unedited Western blots for EV characterisation. 8

Supplementary Fig. 2: Experimental overview and treatment exposure schedule 10

Supplementary Fig. 3: Cell viability after treatment. 11

Supplementary Fig. 4: Representative cut-off frequency and cell layer resistance vs time plots for all conditions tested. 12

Supplementary Fig. 5: Modelling the transient drug response using electrical measurements. 13

Supplementary Fig. 6: Linear regression of MDA-TEV treatment condition. 14

Supplementary Fig. 7: IF imaging of MDA-TEV and TGF-β1 treated MCF10A cells. 16

Supplementary Fig. 8: Unedited x/z and y/z orthogonal views of each cell layer in the OECT channel. 17

Supplementary Fig. 9: Unedited immunoblots of whole-cell lysates collected on treatment day 9 against EMT markers. 18

Supplementary Fig. 10: MDA-TEVs modulate TWIST1 protein abundance but not DNA methylation level. 20

Supplementary Fig. 11: Unedited immunoblots of TWIST1 protein abundance. 22

Supplementary Fig. 12: OECT-based measurements of heparin treatment. 23

SUPPLEMENTARY DISCUSSIONS

Supplementary Discussion 1: A model to recapitulate TEV-driven invasive ductal carcinoma. 5

Supplementary Discussion 2: Investigating EMT requires high temporal resolution and functional readouts 8

Supplementary Discussion 3: Molecular mechanisms of EMT and determining its occurrence 14

Supplementary discussion 4: MDA-TEVs do not influence TWIST1 and TFPI2 DNA methylation levels 18

METHODS AND MATERIALS

DNA extraction and methylation qPCR

QIAamp DNA mini kit (Qiagen) was used to extract DNA from TEV-treated un-treated MCF10A cells according to the manufacturer's protocol. A total DNA input of 300 ng was bisulfite converted using the EZ DNA Methylation Gold kit (Zymo) following manufacturer's protocol. For the qPCR assay, a TaqMan approach was used involving gene-specific primers and probe targeting the methylated sequences. No amplification detected was considered to be non-methylated DNA for the target. Each sample was run in triplicate for the two genes; TWIST1 and TFPI2, and for the house-keeper gene B-actin used to control for variances in DNA input. A standard curve was initially established using fully methylated DNA (Merck), and each subsequent qPCR run included a calibrator (1:100 dilution) to allow for quantification and negative controls.

Each PCR contained a final concentration of 1x LightCycler Probes (Roche), 0.6 µM each primer, 0.2 µM probe, 2.5% DMSO, 1M betaine and 2 µL bisulfite converted DNA in a final reaction volume of 10 µL. Reactions were carried out using a LightCycler 480II (Roche) using the following qPCR thermal cycling conditions; 95°C for 10 mins, 50 cycles of 95°C for 15 sec, 60 °C for 60 sec including data acquisition. Methylation was determined as previously described by Chettouh et al., (2018):

$$Total\ Methylation\ (\%) = \sum \frac{(\frac{A}{B})}{(\frac{C}{D})} \qquad (4)$$

A= methylation value of gene of interest in each sample; B = methylation value of the gene of interest in the calibrator; C= amplification value of β actin in each sample; and D= amplification value of β actin in the calibrator.

Sequence Name	Nucleotide Sequence

TWIST1_Forward	CGTCTACAACTCCTCGTAAAACTACG
TWIST1_Reverse	TCGGGTAGTTCGGTTTAGGGTAAG
TWIST1_Probe	[6FAM]ACTCCCGCCGCCGCTACTACTACC[TAM]
TFPI2_Forward	TGTAGGGGGTCGGGCGGTTC
TFPI2_Reverse	CGCTCGCCCCGCATAAAACG
TFPI2_Probe	[6FAM]CGTTTGGCGGGAGGAGGTGCGCGGTT[TAM]
Beta-actin_Forward	TGGTGATGGAGGAGGTTTAGTAAGT
Beta-actin Reverse	AACCAATAAAACCTACTCCTCCCTTAA
Beta-actin_Probe	[6FAM]ACCACCACCCAACACACAATAACAAACACA[TAM]

Supplementary Discussion 1: A model to recapitulate TEV-driven invasive ductal carcinoma.

It is now well established that TEVs provide autocrine and paracrine signals within the tumour ecosystem to activate an EMT programme in neoplastic epithelial cells[1,2]. This endows the recipient cells with the ability to invade the tissue surrounding the primary tumour, intravasate, and enter the circulation[3] (Fig. 1). TEVs accomplish this by transferring functional cargo comprising transcriptional regulators[4], EMT drivers[1,5], and signalling molecules[6] that influences signalling pathways, such as the canonical Wnt/β-catenin pathway[7], and alters the transcriptome and proteome of recipient cells, thereby inducing a change in cell phenotype[7]. TEVs derived from TNBC cells were reported to promote proliferation and drug resistance in non-tumourigenic breast cells[8], and TEVs from young women's breast cancer patients were found to drive increased invasion of non-malignant cells[9]. In breast carcinomas, the proclivity for mesenchymal transition may be related to the high aggressiveness and characteristic metastatic spread of these tumours[10]. TNBC is a particularly aggressive and invasive breast cancer subtype[11] with metastases frequently occurring in the first 3 years following surgery and a very low 5-year survival rate for afflicted patients (77% compared to 93% for other breast cancer subtypes[12]). The higher mortality is partly due to a disproportionate number of metastatic disease cases[13]. Treatment of TNBC has been limited due to the lack of well-defined therapeutic targets[14] and the scarcity of effective targeted therapies is in part to blame for a poorer prognosis and treatment outcome for TNBC than other types of breast cancer[11]. Studies of progression from ductal carcinoma *in situ* to invasive disease have been facilitated by the use of the MCF10A series[4,15], which have intrinsic phenotypic plasticity for mesenchymal transition[10] and are widely used for investigating EMT in premalignant cells[16,17]. MCF10A cells display characteristics of luminal ductal cells but not of myoepithelial cells (Fig. 1) and they make up the mammary ductal microenvironment of the terminal ductal

lobular unit, which is the origin of most pathologic breast lesions[18]. By contrast, MDA-MB-231 is a cancerous epithelial cell line isolated from a patient with metastatic mammary adenocarcinoma; a type of cancer that begins in the glandular tissue of breast lobules and ducts. Therefore, inducing EMT in MCF10A cells by exposure to MDA-MB-231-derived TEVs (MDA-TEVs) represents a highly relevant disease model for investigating the lesions preceding invasive breast cancer. There is ample precedent for using it, as TEVs derived from MDA-MB-231 cells stimulated with linoleic acid[19] or insulin-like growth factor-1[20] were found to mediate EMT in MCF10A cells, as did hypoxic small EVs derived from the same cell line[21].

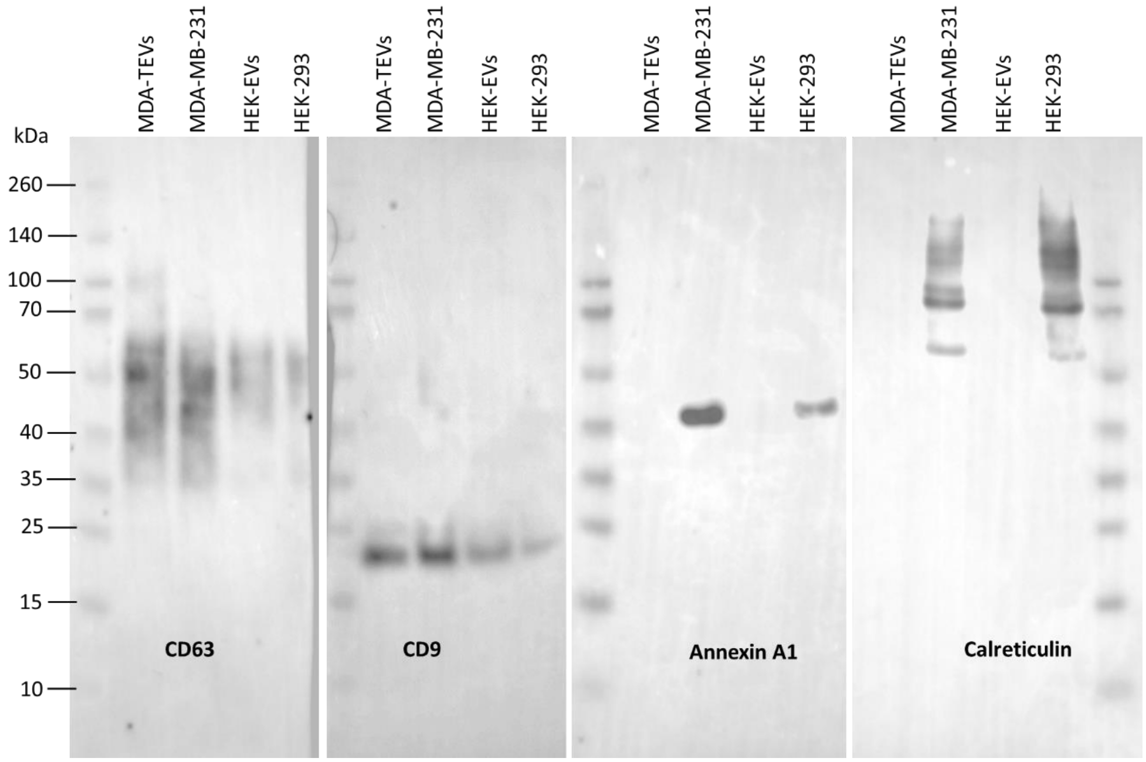

Supplementary Fig. 1: Unedited Western blots for EV characterisation. Samples probed for EV markers CD63 and CD9, microvesicle marker annexin A1, and negative marker calreticulin.

Supplementary Discussion 2: Investigating EMT requires high temporal resolution and functional readouts

Our platform aims to address the limitations of current sensing technologies in metastasis research. Namely, the inability to monitor the transient state of a dynamic biological system and produce quantitative, time-series data relevant to the biology at play. EMT is a transient process and one of the difficulties in its study arises because the transitions between epithelial and mesenchymal states are not binary[22]. Carcinoma cells often exhibit a spectrum of epithelial-mesenchymal characteristics[23,24] and studies have shown that cancer cells, including breast cancers, express both epithelial and mesenchymal markers[25,26]. Therefore, the existence of hybrid EMT states or phenotypes (partial EMT) have been suggested to describe a tumour cell differentiation state in which cancer cells keep both EMT and mesenchymal-to-epithelial transition (MET; reverse-EMT) characteristics. This may promote tumour cell plasticity and tumour progression[25,27,28] and is thus an important feature to capture during phenotype screening. Moreover, migration of cells does not necessarily require the cells to lose all epithelial features, as epithelial cells can migrate as single cells or collectively, while attached with one another via weakened cell-cell interactions[29]. This indicates that the migratory and invasive capacity of cells undergoing EMT is not accurately represented by the simple expression, or lack of expression, of selected (mesenchymal) genes. As such, functional changes in the biological properties of cells must be assessed as well[30].

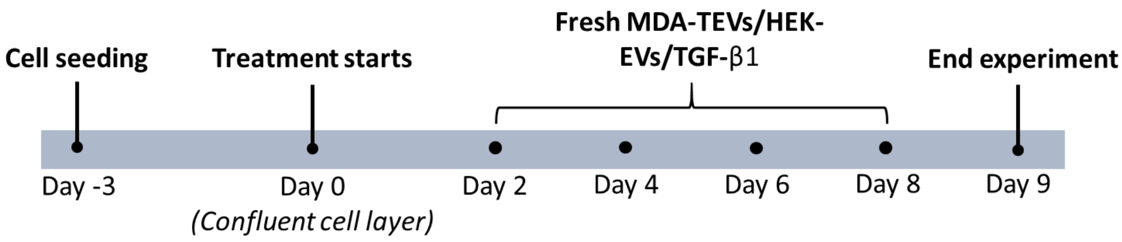

Supplementary Fig. 2: Experimental overview and treatment exposure schedule

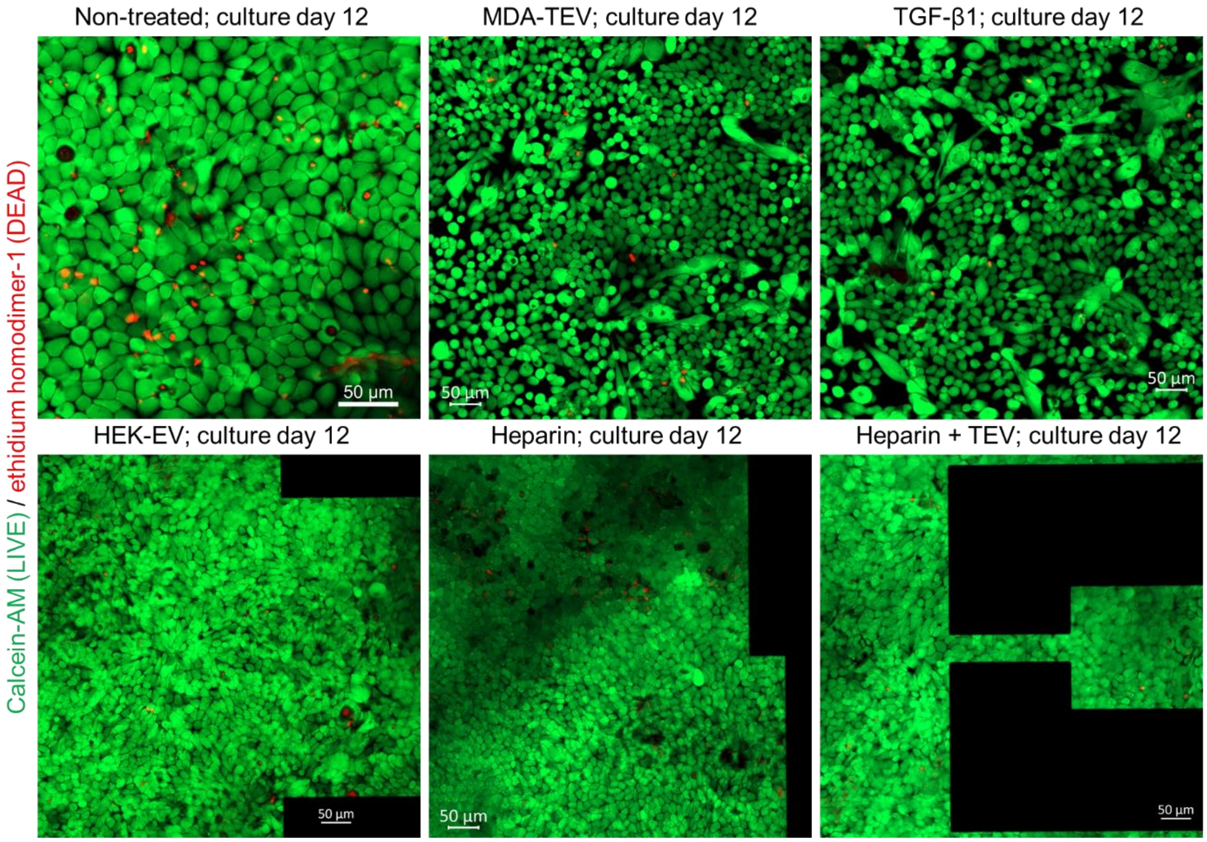

Supplementary Fig. 3: Cell viability after treatment. Cells cultured for 12 days on OECTs with and without exposure as indicated in the figure stained with viability/cytotoxicity kit probes (green-LIVE: Calcein AM; red-DEAD: ethidium homodimer-1).

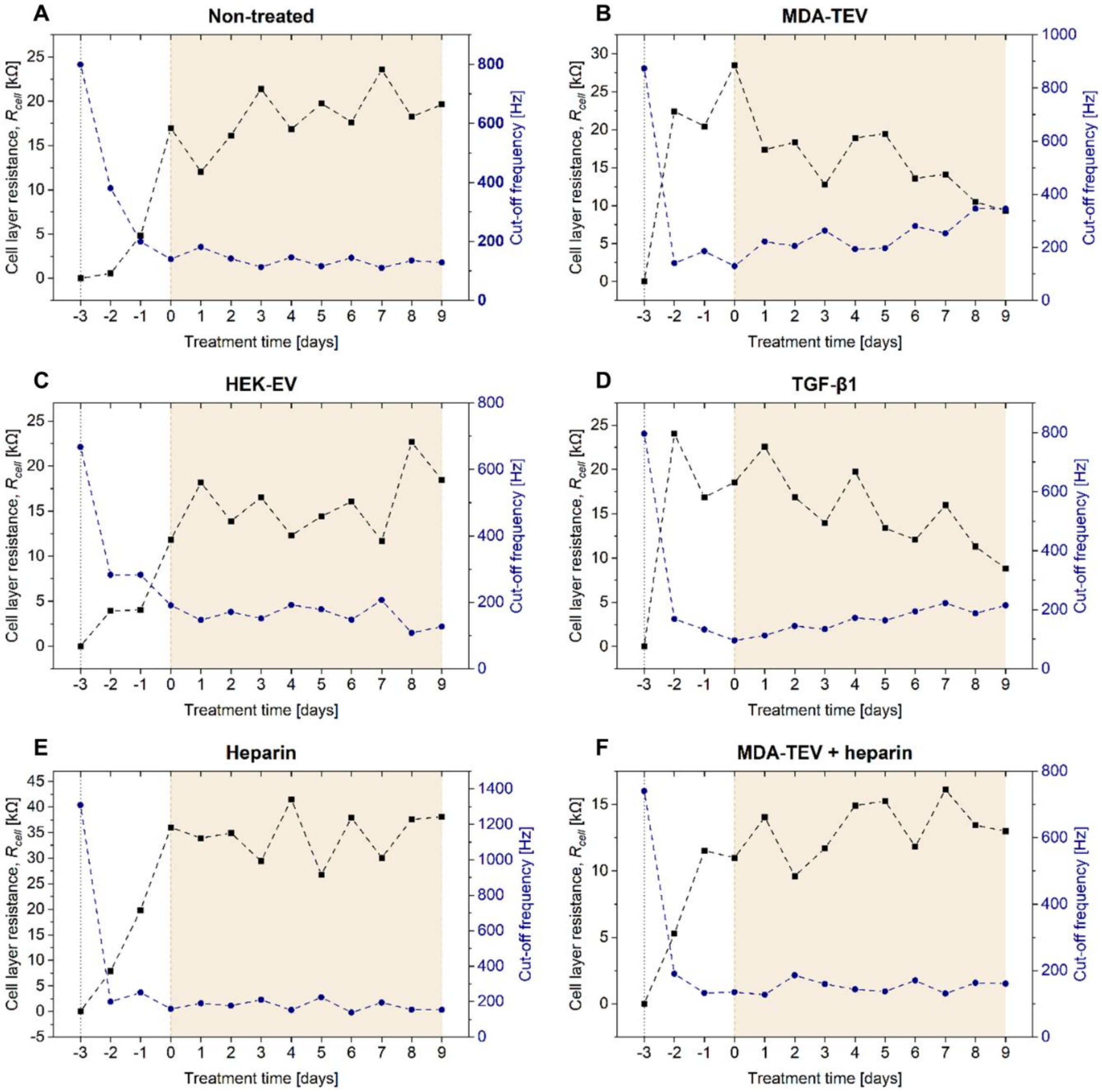

Supplementary Fig. 4: Representative cut-off frequency and cell layer resistance vs time plots for all conditions tested. (**A**) Non-treated. (**B**) MDA-TEV treated. (**C**) HEK-EV treated. (**D**) TGF-β1 treated. (**E**) Heparin treated. (**F**) MDA-TEV + heparin treated.

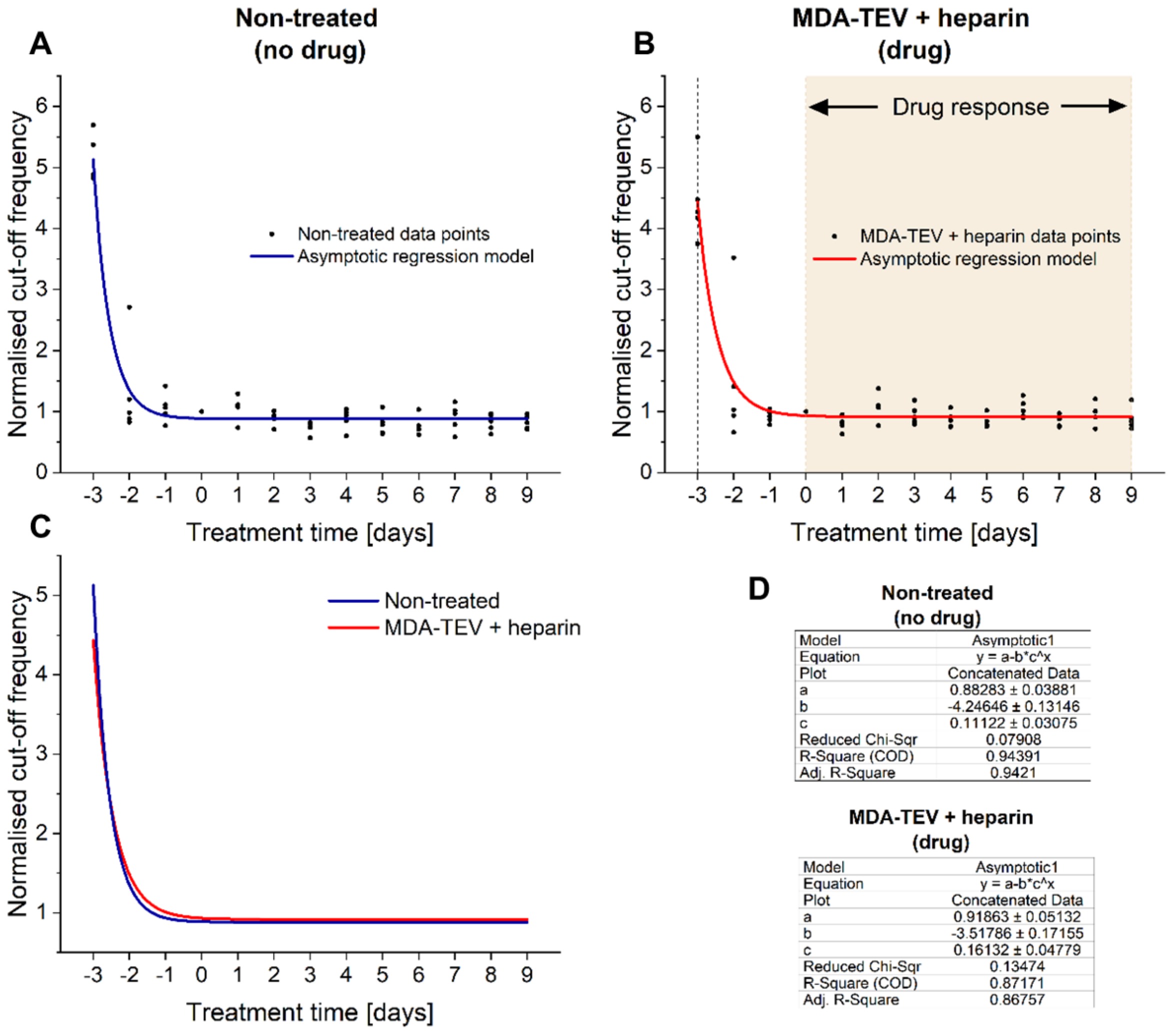

Supplementary Fig. 5: Modelling the transient drug response using electrical measurements. Asymptotic regression of non-treated (**A**) and MDA-TEV + heparin treated (**B)** MCF10A cells, demonstrating that the normalised cut-off frequency tends towards 0.88 $\pm$ 0.034 and 0.92 $\pm$ 0.051, respectively. (**C, D**) The two models are very similar, indicating that heparin treatment of cells exposed to MDA-TEVs block any malignant transformation.

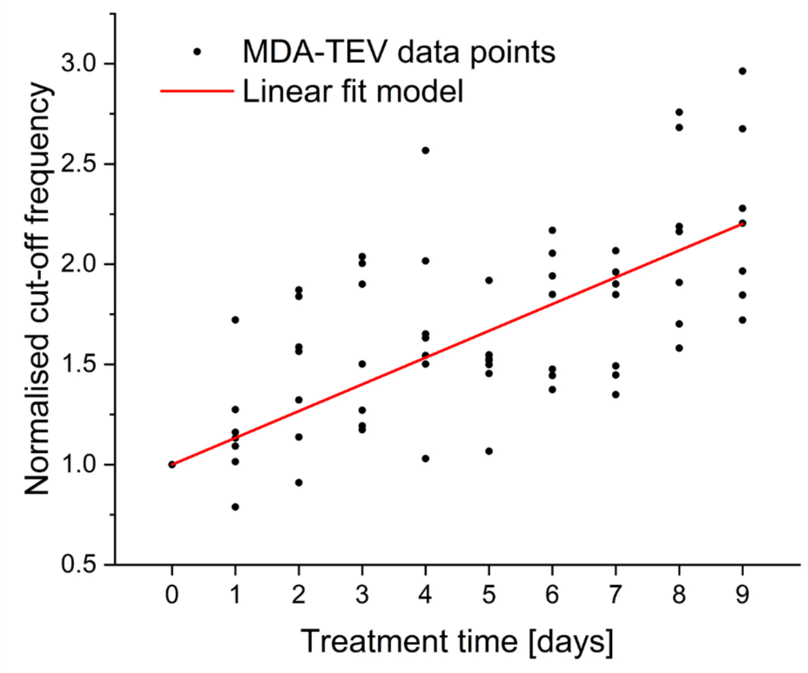

Supplementary Fig. 6: Linear regression of MDA-TEV treatment condition.

Supplementary Discussion 3: Molecular mechanisms of EMT and determining its occurrence

EMT status is typically defined by changes in cellular properties together with a set of molecular markers[30]. Epithelial cells are connected to one another via a variety of epithelial cell junctions that help maintain epithelial polarity, while mesenchymal cells do not contain functional epithelial junctions and present a back-front polarity in their actin stress fibres, characteristic of a migrating cell[30]. During EMT, epithelial cells lose their apical-basal polarity and lateral cell–cell adhesion and gain migratory and invasive properties. Loss of apico-basal polarity leads to destabilisation of adhesion complexes, such as tight junctions and adherens junctions at the lateral membrane[31], and is preceded by reorganisation of filamentous actin (F-actin) (Fig. 1). Actin filaments in mesenchymal cells are bundled into thick contractile stress fibres that increase cellular contractability for migration[32]. Vimentin, a type III intermediate filament found in mesenchymal cells, mediates cytoskeletal organisation and focal adhesion maturation, which the migrating cells utilise to attach to the ECM[33]. Previous studies using the same MDA-TEV-EMT model[19,20] assessed transcript expression and protein abundance using RT-qPCR and Western blot and demonstrated that MDA-TEVs induced down-regulation of *E-cadherin* (epithelial marker) expression and upregulation of *Twist-related protein 1* (TWIST1) transcripts, as well as *vimentin* and *N-cadherin* (mesenchymal markers) expression in MCF10A cells. TWIST1 is an E-cadherin transcriptional repressor that binds to promoter E-boxes on E-cadherin to repress transcription and they are part of the canonical Wnt/β-catenin pathway, indicating that TEVs (and their cargo) modulate this EMT pathway. Functionally, TEVs promoted migration and invasion in MCF10A cells, as measured by migration/wound-healing and Boyden chamber assays[19,20].

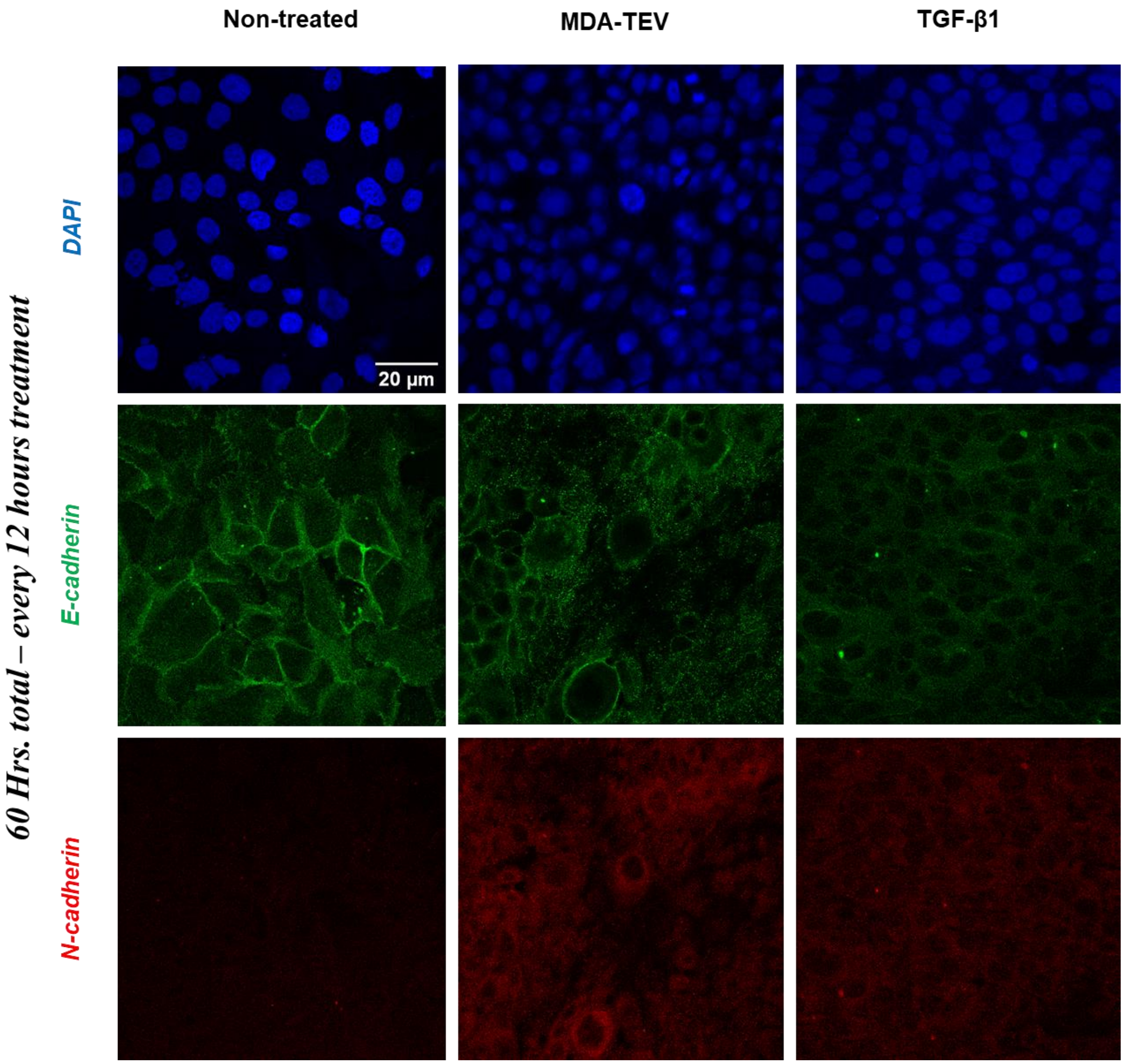

Supplementary Fig. 7: IF imaging of MDA-TEV and TGF-β1 treated MCF10A cells. E-cadherin (green), N-cadherin (red), and nuclei (blue) on MCF10A monolayer with no treatment (left column), treated with 50 μg of MDA-TEVs (middle column), or with 10 ng/mL TGFβ (right column) every 12 hours for 60 hours.

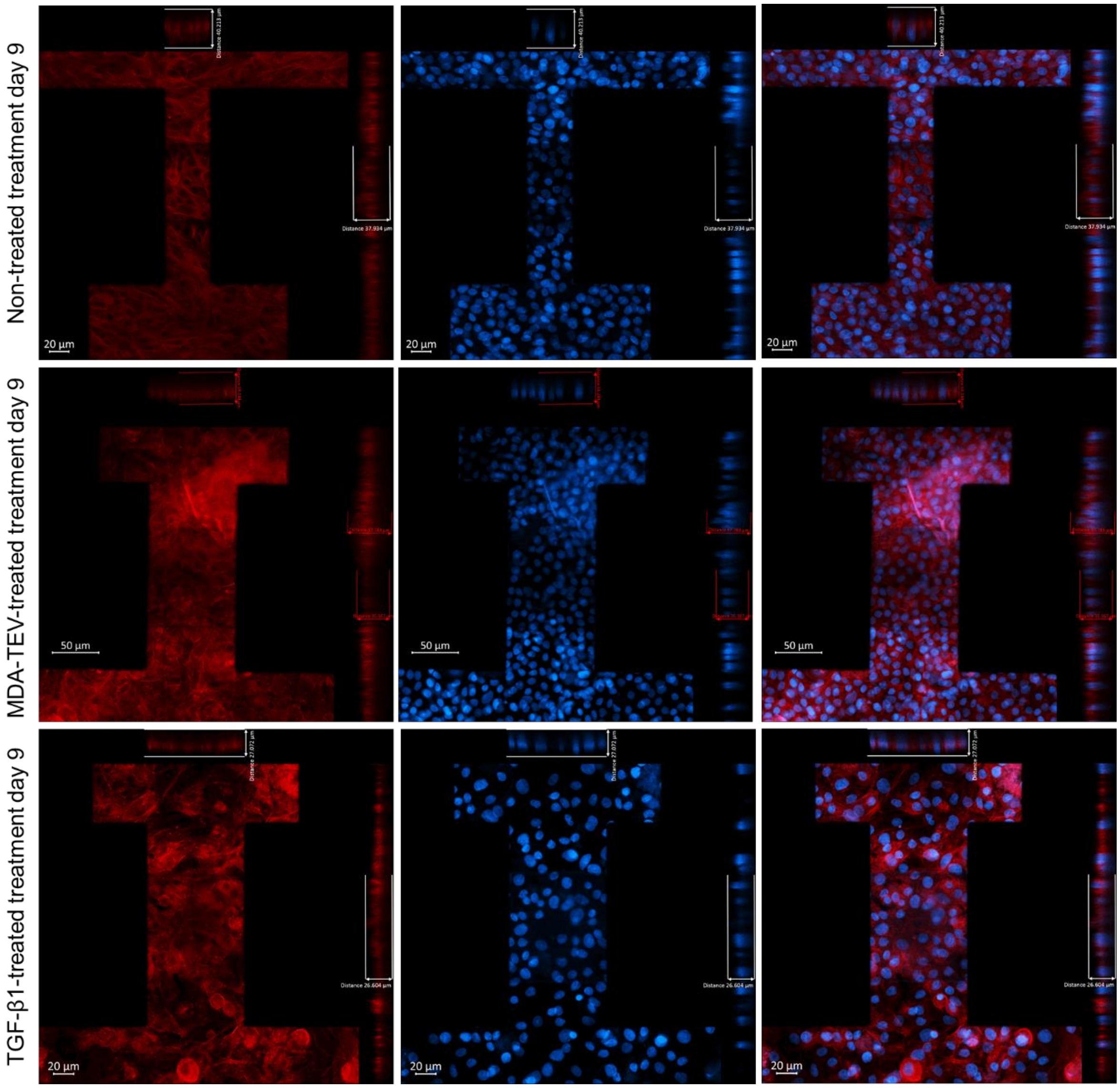

Supplementary Fig. 8: Unedited x/z and y/z orthogonal views of each cell layer in the OECT channel. Imaging performed on MDA-TEV treatment day 9 and obtained by z-stacked confocal images. Cell height was calculated using the built-in tools in the Zen software, as shown in the images.

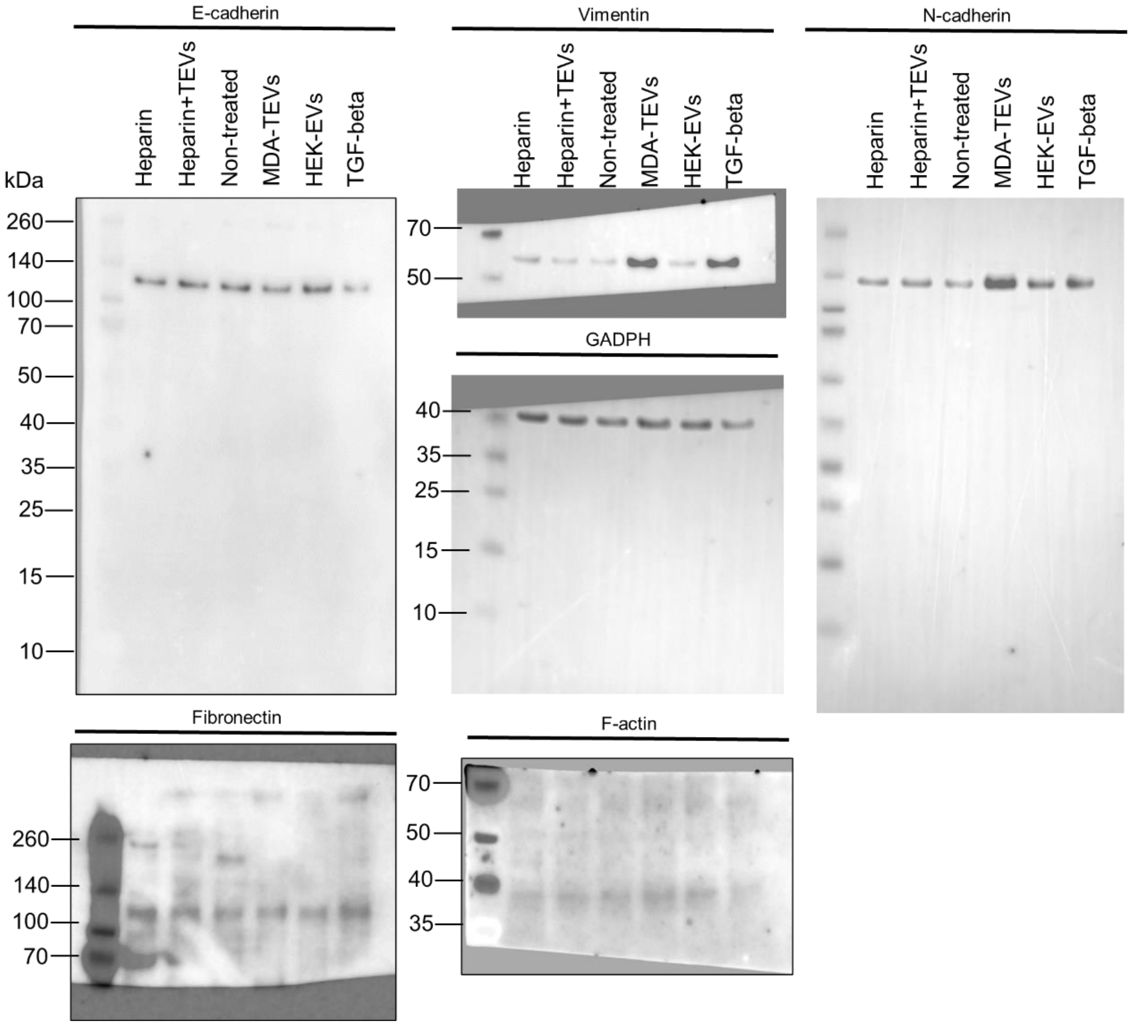

Supplementary Fig. 9: Unedited immunoblots of whole-cell lysates collected on treatment day 9 against EMT markers. Equal quantities of protein were separated on SDS-PAGE gels and membranes were blotted with indicated antibodies. E-cadherin, predicted: 97 kDa; Vimentin, predicted: 54-75 kDa; N-cadherin, predicted: 125-135 kDa; fibronectin (slightly smeared due to glycosylation[34] and shows multiple bands with lower MW;), predicted: 262-285 kDa; and F-actin (band at ~70 kDa may indicate presence of dimers), predicted 42 kDa. The presence of multiple bands is also indicated on antibody product page (abcam).

Supplementary discussion 4: MDA-TEVs do not influence TWIST1 and TFPI2 DNA methylation levels

We sought to gain mechanistic insight into the mode of action (MoA) of MDA-TEV-induced EMT via epigenetics analysis. Epigenetic remodelling is prevalent during breast cancer metastasis[35] and DNA methylation of tumour and metastasis suppressor genes is a hallmark of circulating tumour cells[36]. Gene promoters, especially key tumour suppressor genes, are unmethylated in normal tissues and highly methylated in cancer tissues[37]. Gene promoter hypermethylation is thus a pathway for repression of gene transcription (transcriptional silencing), and conversely, gene promotor hypomethylation (loss of DNA methylation) may promote gene expression[38]. Oncogene BCR-ABL1-positive EVs released from leukaemia cells have been demonstrated to increase global DNA methylation levels in recipient cells[39]. We sought to determine whether MDA-TEVs influence DNA methylation by analysing the methylation status of the tumour suppressor gene *tissue factor pathway inhibitor 2* (*TFPI2*) and the pro-metastatic transcription factor *TWIST1*. TWIST1 represses E-cadherin and promotes EMT[19] and *TWIST1* transcripts were reportedly upregulated after MDA-TEV treatment[19,20]. TWIST1 is negatively associated with TFPI2 in breast cancer patients, as TFPI2 suppresses breast cancer progression through inhibiting TWIST-integrin α5 pathway[40]. TFPI2 is downregulated in breast cancer cells lines compared to MCF10A cells and methylation in the *TFPI2* promotor has been found in highly invasive breast cancer cells[41]. We observed upregulation of TWIST1 protein between MDA-TEV and non-treatment conditions (Supplementary Fig. 10a), which likely caused E-cadherin repression (Fig. 4g,h). However, MDA-TEVs do not appear to carry TWIST1 (Supplementary Fig. 10b).

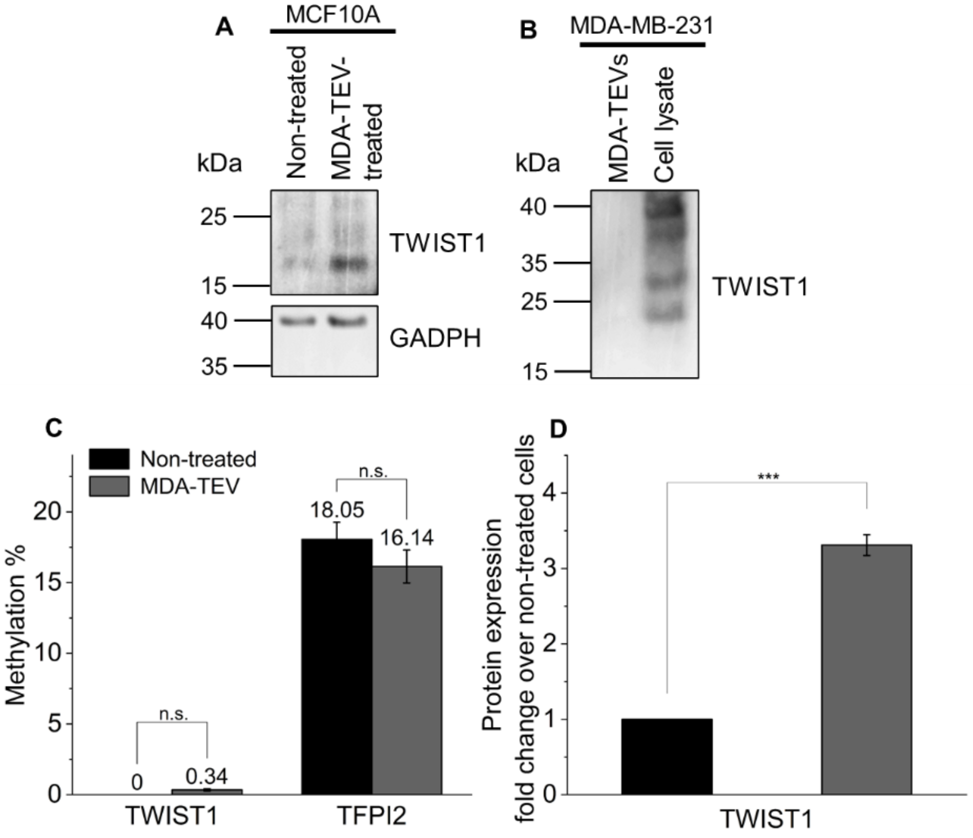

Supplementary Fig. 10: MDA-TEVs modulate TWIST1 protein abundance but not DNA methylation level. (**A**) Immunoblots of whole-cell lysates collected on treatment day 9. (**B**) Immunoblot of MDA-TEVs and MDA-MB-231 whole-cell lysate. Equal quantities of protein were separated on SDS-PAGE gels and membranes were blotted with indicated antibodies. TWIST1, predicted: 21 kDa; GADPH, predicted: 37 kDa. Unedited blots are available in Supplementary Fig. 11. (**C**) Total methylation (%) relative to house-keeping gene *β-actin* (mean ± s.e.m.; n=1). (**D**) Quantitative values of protein expression derived from immunoblots presented as fold-change versus non-treated expression level (mean ± s.e.m.; n=3). *** p≤0.001, n.s. = non-significant.

A MethyLight-based approach was used to determine the methylation of these markers [42]. This was based on previous work published by Chettouh et al (2018), which looked at markers of Barrett's oesophagus for methylation[43]. We found that neither *TFPI2* nor *TWIST1* was significantly differentially methylated between non-treated and MDA-TEV-treated cells (Supplementary Fig. 10c). Surprisingly, *TFPI2* displayed higher levels of methylation compared to *TWIST1* across both conditions. This raises the question of whether *TFPI2* (and

TWIST1) methylation status in MCF10A cells could potentially contribute to the cell line's intrinsic phenotypic plasticity for mesenchymal transition.

Interestingly, immunoblot analysis revealed that TWIST1 protein was present in different amounts between the non-treatment and MDA-TEV treatment conditions (Supplementary Fig. 10 a,d), which could suggest that *TWIST1* is being repressed post-transcriptionally in non-treated MCF10A cells, e.g. by CPEB1/2 and miR-580[44]. miR-580 acts as a negative regulator of *TWIST1* expression in MCF10A cells and is downregulated in MCF10A cells which have undergone EMT[44]. As MDA-TEVs increase the abundance of TWIST1 protein, *TWIST1* could be upregulated post-transcriptionally by other regulatory mechanisms, e.g. MDA-TEV-delivered miR-580-siliencing circular RNAs[45], or even be induced by upregulation of transcriptional factors, e.g. HMGA2[46]. Further immunoblot analysis revealed that TWIST1 was not part of MDA-TEV cargo, although highly abundant in MDA-MB-231 cells (Supplementary Fig. 10 b). These results collectively indicate that MDA-TEVs are dysregulating endogenous TWIST1-repression in MCF10A cells via mechanisms other than epigenetic remodelling or direct protein transfer.

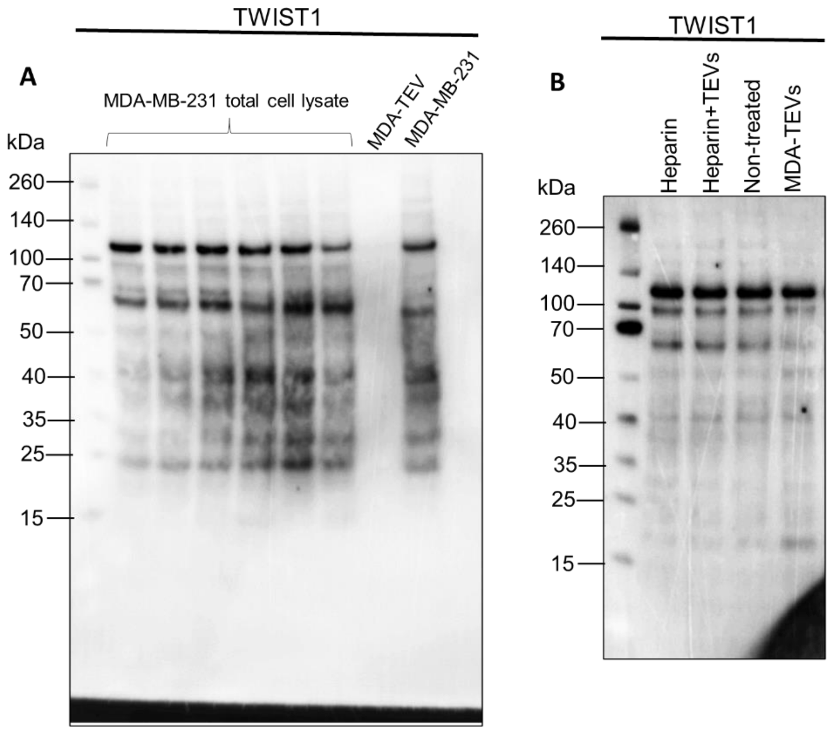

Supplementary Fig. 11: Unedited immunoblots of TWIST1 protein abundance. (**A**) MDA-MB-231 total cell lysate and MDA-TEVs. (**B**) MCF10A cells treated under conditions indicated. TWIST1 has a predicted molecular weight of 21 kDa. The presence of multiple bands is also indicated on antibody product page (abcam).

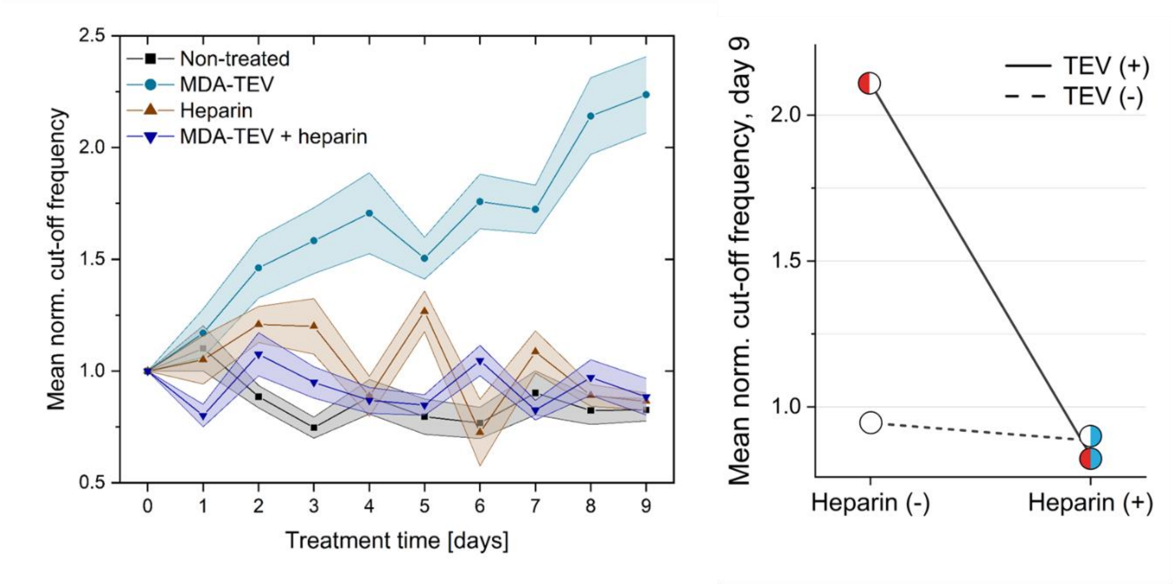

Supplementary Fig. 12: OECT-based measurements of heparin treatment. LHS: Cut-off frequency normalised to day 0 (treatment start day) over time for the four treatment conditions: non-treated; 200 µg MDA-TEVs; 10 µg/ml heparin; and 200 µg MDA-TEVs + 10 µg/ml heparin, (mean ± s.e.m.; s.e.m. indicated by lightly coloured areas; n=3). Data points before treatment day 0 are omitted for clarity. RHS: Interaction plot illustrating the relationship between MDA-TEV and heparin treatment (mean; n=3).

References and Notes

1. Aga, M. *et al.* Exosomal HIF1α supports invasive potential of nasopharyngeal carcinoma-associated LMP1-positive exosomes. *Oncogene* **33**, 4613–4622 (2014).
2. Franzen, C. A. *et al.* Urothelial cells undergo epithelial-to-mesenchymal transition after exposure to muscle invasive bladder cancer exosomes. *Oncogenesis* **4**, e163–e163 (2015).
3. Syn, N., Wang, L., Sethi, G., Thiery, J.-P. & Goh, B.-C. Exosome-Mediated Metastasis: From Epithelial–Mesenchymal Transition to Escape from Immunosurveillance. *Trends Pharmacol. Sci.* **37**, 606–617 (2016).
4. Melo, S. A. *et al.* Cancer Exosomes Perform Cell-Independent MicroRNA Biogenesis and Promote Tumorigenesis. *Cancer Cell* **26**, 707–721 (2014).
5. You, Y. *et al.* Matrix metalloproteinase 13-containing exosomes promote nasopharyngeal carcinoma metastasis. *Cancer Sci.* **106**, 1669–1677 (2015).
6. Ramteke, A. *et al.* Exosomes secreted under hypoxia enhance invasiveness and stemness of prostate cancer cells by targeting adherens junction molecules. *Mol. Carcinog.* **54**, 554–565 (2015).
7. Chen, Y. *et al.* Aberrant low expression of p85α in stromal fibroblasts promotes breast cancer cell metastasis through exosome-mediated paracrine Wnt10b. *Oncogene* **36**, 4692–4705 (2017).
8. Ozawa, P. M. M. *et al.* Extracellular vesicles from triple-negative breast cancer cells promote proliferation and drug resistance in non-tumorigenic breast cells. *Breast Cancer Res. Treat.* **172**, 713–723 (2018).
9. Jordan, K. R. *et al.* Extracellular vesicles from young women's breast cancer patients drive increased invasion of non-malignant cells via the Focal Adhesion Kinase pathway: a proteomic approach. *Breast Cancer Res.* **22**, 128 (2020).

10. Sarrió, D. *et al.* Epithelial-Mesenchymal Transition in Breast Cancer Relates to the Basal-like Phenotype. *Cancer Res.* **68**, 989–997 (2008).

11. Goh, C. Y. *et al.* Exosomes in triple negative breast cancer: Garbage disposals or Trojan horses? *Cancer Lett.* **473**, 90–97 (2020).

12. Bauer, K. R., Brown, M., Cress, R. D., Parise, C. A. & Caggiano, V. Descriptive analysis of estrogen receptor (ER)-negative, progesterone receptor (PR)-negative, and HER2-negative invasive breast cancer, the so-called triple-negative phenotype. *Cancer* **109**, 1721–1728 (2007).

13. Fulford, L. G. *et al.* Basal-like grade III invasive ductal carcinoma of the breast: patterns of metastasis and long-term survival. *Breast Cancer Res.* **9**, R4 (2007).

14. Yao, H. *et al.* Triple-negative breast cancer: is there a treatment on the horizon? *Oncotarget* **8**, 1913–1924 (2016).

15. Santner, S. J. *et al.* Malignant MCF10CA1 Cell Lines Derived from Premalignant Human Breast Epithelial MCF10AT Cells. *Breast Cancer Res. Treat.* **65**, 101–110 (2001).

16. Qu, Y. *et al.* Evaluation of MCF10A as a Reliable Model for Normal Human Mammary Epithelial Cells. *PLOS ONE* **10**, e0131285 (2015).

17. Puleo, J. & Polyak, K. The MCF10 Model of Breast Tumor Progression. *Cancer Res.* **81**, 4183–4185 (2021).

18. Cichon, M. A., Degnim, A. C., Visscher, D. W. & Radisky, D. C. Microenvironmental Influences that Drive Progression from Benign Breast Disease to Invasive Breast Cancer. *J. Mammary Gland Biol. Neoplasia* **15**, 389–397 (2010).

19. Galindo-Hernandez, O., Serna-Marquez, N., Castillo-Sanchez, R. & Salazar, E. P. Extracellular vesicles from MDA-MB-231 breast cancer cells stimulated with linoleic acid promote an EMT-like process in MCF10A cells. *Prostaglandins Leukot. Essent. Fatty Acids* **91**, 299–310 (2014).

20. Leal-Orta, E., Ramirez-Ricardo, J., Garcia-Hernandez, A., Cortes-Reynosa, P. & Salazar, E. P. Extracellular vesicles from MDA-MB-231 breast cancer cells stimulated with insulin-like growth factor 1 mediate an epithelial–mesenchymal transition process in MCF10A mammary epithelial cells. *J. Cell Commun. Signal.* (2021) doi:10.1007/s12079-021-00638-y.

21. Bertolini, I., Perego, M., Ghosh, J. C., Kossenkov, A. V. & Altieri, D. C. NFκB activation by hypoxic small extracellular vesicles drives oncogenic reprogramming in a breast cancer microenvironment. *Oncogene* **41**, 2520–2525 (2022).

22. Lai, X. *et al.* Epithelial-Mesenchymal Transition and Metabolic Switching in Cancer: Lessons From Somatic Cell Reprogramming. *Front. Cell Dev. Biol.* **8**, (2020).

23. Zhang, Y. & Weinberg, R. A. Epithelial-to-mesenchymal transition in cancer: complexity and opportunities. *Front. Med.* **12**, 361–373 (2018).

24. Jolly, M. K., Ware, K. E., Gilja, S., Somarelli, J. A. & Levine, H. EMT and MET: necessary or permissive for metastasis? *Mol. Oncol.* **11**, 755–769 (2017).

25. Hiew, M. S. Y. *et al.* Incomplete cellular reprogramming of colorectal cancer cells elicits an epithelial/mesenchymal hybrid phenotype. *J. Biomed. Sci.* **25**, 57 (2018).

26. Bronsert, P. *et al.* Cancer cell invasion and EMT marker expression: a three-dimensional study of the human cancer–host interface. *J. Pathol.* **234**, 410–422 (2014).

27. Sampson, V. B. *et al.* Wilms' Tumor Protein Induces an Epithelial-Mesenchymal Hybrid Differentiation State in Clear Cell Renal Cell Carcinoma. *PLOS ONE* **9**, e102041 (2014).

28. Schliekelman, M. J. *et al.* Molecular Portraits of Epithelial, Mesenchymal, and Hybrid States in Lung Adenocarcinoma and Their Relevance to Survival. *Cancer Res.* **75**, 1789–1800 (2015).

29. Wrenn, E. D. *et al.* Regulation of Collective Metastasis by Nanolumenal Signaling. *Cell* **183**, 395-410.e19 (2020).

30. Yang, J. *et al.* Guidelines and definitions for research on epithelial–mesenchymal transition. *Nat. Rev. Mol. Cell Biol.* **21**, 341–352 (2020).
31. Barrios-Rodiles, M. *et al.* High-Throughput Mapping of a Dynamic Signaling Network in Mammalian Cells. *Science* **307**, 1621–1625 (2005).
32. Haynes, J., Srivastava, J., Madson, N., Wittmann, T. & Barber, D. L. Dynamic actin remodeling during epithelial–mesenchymal transition depends on increased moesin expression. *Mol. Biol. Cell* **22**, 4750–4764 (2011).
33. Liu, C.-Y., Lin, H.-H., Tang, M.-J. & Wang, Y.-K. Vimentin contributes to epithelial-mesenchymal transition cancer cell mechanics by mediating cytoskeletal organization and focal adhesion maturation. *Oncotarget* **6**, 15966–15983 (2015).
34. Verma, R. Fibronectin. in *Encyclopedia of Cancer* (ed. Schwab, M.) 1399–1402 (Springer, 2011). doi:10.1007/978-3-642-16483-5_2182.
35. Reyngold, M. *et al.* Remodeling of the Methylation Landscape in Breast Cancer Metastasis. *PLOS ONE* **9**, e103896 (2014).
36. Chimonidou, M. *et al.* DNA Methylation of Tumor Suppressor and Metastasis Suppressor Genes in Circulating Tumor Cells. *Clin. Chem.* **57**, 1169–1177 (2011).
37. Baylin, S. B. & Jones, P. A. A decade of exploring the cancer epigenome — biological and translational implications. *Nat. Rev. Cancer* **11**, 726–734 (2011).
38. Cheng, Y. *et al.* Targeting epigenetic regulators for cancer therapy: mechanisms and advances in clinical trials. *Signal Transduct. Target. Ther.* **4**, 1–39 (2019).
39. Zhu, X. *et al.* BCR-ABL1–positive microvesicles transform normal hematopoietic transplants through genomic instability: implications for donor cell leukemia. *Leukemia* **28**, 1666–1675 (2014).
40. Zhao, D. *et al.* TFPI2 suppresses breast cancer progression through inhibiting TWIST-integrin α5 pathway. *Mol. Med.* **26**, 27 (2020).

41. Guo, H. *et al.* Tissue factor pathway inhibitor-2 was repressed by CpG hypermethylation through inhibition of KLF6 binding in highly invasive breast cancer cells. *BMC Mol. Biol.* **8**, 110 (2007).
42. Eads, C. A. *et al.* MethyLight: a high-throughput assay to measure DNA methylation. *Nucleic Acids Res.* **28**, e32-00 (2000).
43. Chettouh, H. *et al.* Methylation panel is a diagnostic biomarker for Barrett's oesophagus in endoscopic biopsies and non-endoscopic cytology specimens. *Gut* **67**, 1942–1949 (2018).
44. Nairismägi, M.-L. *et al.* Translational control of TWIST1 expression in MCF-10A cell lines recapitulating breast cancer progression. *Oncogene* **31**, 4960–4966 (2012).
45. Zhan, Y. *et al.* Carcinoma-associated fibroblasts derived exosomes modulate breast cancer cell stemness through exonic circHIF1A by miR-580-5p in hypoxic stress. *Cell Death Discov.* **7**, 1–15 (2021).
46. Tan, E.-J. *et al.* Regulation of transcription factor Twist expression by the DNA architectural protein high mobility group A2 during epithelial-to-mesenchymal transition. *J. Biol. Chem.* **287**, 7134–7145 (2012).
